# Metabolomics and 16S rRNA Gene Sequencing Analyses of Changes in the Intestinal Flora and Biomarkers Induced by *Gastrodia-Uncaria* Treatment in a Rat Model of Chronic Migraine

**DOI:** 10.3389/fphar.2019.01425

**Published:** 2019-12-17

**Authors:** Zhiqi Wen, Mingzhen He, Chunyan Peng, Yifei Rao, Junmao Li, Zhifeng Li, Lijun Du, Yan Li, Maofu Zhou, Ouyang Hui, Yulin Feng, Shilin Yang

**Affiliations:** ^1^School of Pharmacy, Jiangxi University of Traditional Chinese Medicine, Nanchang, China; ^2^State Key Laboratory of Innovative Drug and Efficient Energy, Jiangxi University of Traditional Chinese Medicine, Nanchang, China; ^3^Laboratory of Molecular Pharmacology and Pharmaceutical Sciences, School of Life Sciences, Tsinghua University, Beijing, China

**Keywords:** migraine, *Gastrodia-Uncaria*, 16S rRNA-seq, plasma metabolomics, pharmacodynamic

## Abstract

Accumulating evidence suggests that natural medicines have notable curative effects on neurological conditions, such as migraine, that are mediated by regulating the gut microbial flora. A natural medicine pair used in traditional Chinese medicine, *Gastrodia elata* Blume and *Uncaria rhynchophylla* (Miq.) Miq. ex Havil. (GU), have shown excellent effect in treating migraine, yet the role of gut microbes in the therapeutic effect of GU in chronic migraine (CMG) is unknown. Here, we performed a 16S rRNA gene sequencing and metabolomics study of the effects of GU in a nitroglycerin (NTG)-induced rat model of CMG. Our results showed that the gut microbial community structure changed significantly and was similar to that of control rats after GU administration in CMG rats. Specifically, GU increased the relative abundance of *Bacteroides* and *Coprococcus* and reduced the abundance of *Prevotella*_1 and *Escherichia*-*Shigella* in CMG rats. The metabolomics profiles of the plasma and ileum contents of CMG rats obtained with an ultra-performance liquid chromatography-mass spectrometer (UPLC-MS) revealed similar biomarkers in both samples, and GU treatment reduced 3-indoxyl sulfate, glutamic acid, *L*-tyrosine, and *L*-arginine levels, and increased 5-HIAA, *L*-tryptophan, and linoleic acid levels in plasma. Correlation analysis showed that the affected bacteria were closely related to amino acid metabolism. Most importantly, GU treatment hardly affected biomarkers in feces samples after inhibiting the activity of gut microbes. Collectively, these findings indicate that structural changes in gut flora are closely related to host metabolism and that regulating the gut microbial community structure and function may be one of the important mechanisms underlying the therapeutic effects of GU in migraine.

## Introduction

Migraine, a severe type of headache, is a common chronic neurovascular disorder with recurrent episodes that seriously affects the lives of patients [Headache Classification Committee of the International Headache Society (IHS), 2013]. Though anti-inflammatory drugs, ergot-type preparations, triptans, and calcitonin gene-related peptide (CGRP) receptor antagonists have shown therapeutic effects on migraine in the clinic (Lipton et al., 2000; Olesen et al., 2004), their clinical use has been limited by their side-effects, such as cardiovascular and gastrointestinal disorders (Katsarava et al., 2018), which calls for a rethink of the therapeutic strategy for migraine. Recently, several studies have shown that disturbances of intestinal flora may be associated with neurological disorders, including migraine headache (Foster et al., 2016; Tang et al., 2019). The dysbiosis of gut flora could affect the brain–gut axis, which would promote neurotransmitter disorders, and this appears to be true for migraine-associated neurotransmitters (Grover and Kashyap, 2014).

Natural products, food supplements, and traditional medicine (TM) have been shown to have therapeutic benefits on migraine (Al-Hashel et al., 2018). *Gastrodia elata* Blume and *Uncaria rhynchophylla* (Miq.) Miq. ex Havil. (GE and UR, [GU]) are the main herbs in the GU decoction, which has shown significant efficacy in vascular headache (Jiang, 2007). The prescription preparation, GU granules, in particular, are commonly used for treating neurological conditions such as migraine (Zhang et al., 2017). Extracts of GE and UR have been reported to exert neuroprotective effects (Xian et al., 2016; Liu et al., 2018); however, the oral absorption bioavailability of GE and UR extract is poor (Ge et al., 2014). Many compounds, in particular, natural products taken orally, reach the intestine and are readily metabolized by gut flora. In general, they are decomposed into metabolites with polarity and lower molecular weight, thus having higher bioavailability. Therefore, to study the mechanism of GU in treating migraine, we need to study the function of intestinal flora and metabolism *in vivo*. In this study, omics synthesis was applied to study the treatment mechanism of GU on migraine (Lionetto et al., 2013; Gasparini et al., 2017).

Recently, several studies have indicated that DNA sequence variants affect the risk of migraine subtypes (Sutherland et al, 2019; van den et al, 2019; Grieco et al, 2018), and 16S rRNA gene sequencing and metabolomics have been used as effective tools for studying disease and drug treatment mechanisms (Estruel-Amades et al., 2019). 16S rRNA gene sequencing studies can accurately identify the structure and metabolic functions of the gut microbe. Metabolomics technology provides a unique and novel technique for disease and pharmacodynamics characterization that facilitates systematic analysis of metabolic pathways and related metabolites (Zhang et al., 2018). In recent years, many diseases and drug interventions have been found to cause changes in the structure of the gut microbial flora. Metabolomics techniques have been extensively used in studies of disease and drug mechanisms. However, the mechanism by which GU affects host plasma metabolism through gut microbes remains to be clarified.

This study aims to investigate the effect of GU administration on intestinal flora and biomarkers in migraine rats and to explore the potential linkage. To this end, we probed the changes in gut microbiota, plasma biomarkers, and intestinal biomarkers after GU administration and investigated the possible links between them. The differences in gut microbial community structure and plasma metabolomics were analyzed by 16S rRNA sequencing technology and LC/MS analysis, respectively. In particular, we conducted validation tests consisting of short-term *in vitro* incubation of feces with the drug to prove the effect of GU on the metabolism of the flora. These studies provide a more comprehensive and detailed understanding of the mechanism by which GU mediates therapeutic effects in NTG-induced chronic migraine by regulating the host gut microbial flora.

## Materials and Methods

### Chemicals and Drugs

HPLC grade methanol and acetonitrile were obtained from Fisher Scientific (Fair Lawn, NJ, USA). Pure distilled water was purchased from Wahaha (Hangzhou, China). HPLC grade formic acid was obtained from Shanghai Aladdin Biochemical Technology Co., Ltd., Shanghai, China. 2-Chloro-L-phenylalanine, which was used as the internal standard (IS), was provided by Shanghai Macklin Biochemistry Company, Shanghai, China. Standard 3-indoxyl sulfate was obtained from Beijing LeBo Biotech Co., Ltd., Beijing, China. *L*-Arginine, glutamic acid, *L*-valine, linoleic acid, *L*-phenylalanine, *L*-tryptophan, and *L*-tyrosine were purchased from Shanghai Yuanye Biotechnology Co. Ltd., Shanghai, China.

### Extraction Methods

The pieces of GE and UR used (purchased from Jiangxi Jiangzhong Chinese Herbal Pieces Co. Ltd.) accorded with the standard in the Chinese Pharmacopoeia. To prepare the GU extract, GU (exact amounts of GE and UR in a weight ratio of 9:12) was extracted by reference to the boiling steps for GU Granules in Volume I of the Pharmacopoeia of the People’s Republic of China (Edition 2015). The extraction process was that herbs were decocted twice with water and filtered, then the decoctions were merged, and the filtrate was concentrated. The detailed method is shown in **Figure S1**. The concentrated extract was dried in a water bath at 60°C. The final yield was powdered and then stored in a desiccator at room temperature. Water extracts of GU were analyzed with a Waters ACQUITY UPLC System (Waters Corp. Milford, USA). The extract was dissolved in water and then filtered through a 0.22-µm filter before being separated in an Acquity UPLC HSS T3 column (100 mm × 2.1 mm, 1.8 µm) at 40°C. The mobile phase was composed of 0.1% formic acid water (A) and acetonitrile (B), and the gradient program was as follows: 0–3 min, 2% B; 3–9 min, 2–12% B; 9–24 min, 12–32% B; 24–29 min, 32–75% B; 29–29.1 min, 75–100% B; 29.1–32 min, 100% B. The flow rate was 0.3 mL/min, and the injection volume was 1 µL. To determine the composition of the compounds in the GU extract, the effluent was introduced into an MS for mass spectrometry analysis, using a Waters SYNAPT G2 system with an electrospray ionization (ESI) source operating in both positive and negative ion modes. Briefly, the parameters of the positive ion mode were set as follows: capillary voltage, 4.0 kV; source temperature, 120°C; cone gas rate, 40 L/h; desolvation gas rate and temperature, 800 L/h and 400°C; cone voltage, 19 V; collision energy 25–40 V; scan time and inter-scan delay, 0.15 and 0.02 s. The parameters used in the negative ion mode were the same as those used in the positive ion mode except: capillary voltage, 2.5 kV; cone voltage, 25 V. The data were collected from m/z 50 to 1,200 Da.

### Animals and Treatments

Specific pathogen-free (SPF) female Sprague–Dawley rats (weight: 220 ± 20 g, purchased from the Hunan Slack Jingda Laboratory Animal Co., Ltd., Hunan, China) were acclimated for 5 days under standard laboratory conditions at Jiangxi University of Traditional Chinese Medicine, with free access to food and water. Subsequently, 60 rats were randomly divided into Control, Model, Sumatriptan, and GU groups. Rats in the Sumatriptan and GU groups were orally administered Sumatriptan (25 mg/Kg/d) or GU (4 g/Kg/d), respectively, for 10 days. The dose was determined by the pre-experiment results. Rats in the Control and Model groups received distilled water orally. At the same time, rats in the Control group rats received saline (intraperitoneal [i.p.] on days 3, 5, 7, and 9), while the other groups were injected with NTG (10 mg/Kg, i.p.) (Farajdokht et al., 2018).

All experimental procedures used in this study were based on the ethical principles for laboratory animals of the State Key Laboratory (Reference number: BCTG-2016-18).

### Sample Collection and Preparation

One hour after the final oral administration, feces samples were collected in sterile conical tubes and stored at -80°C. Subsequently, all rats in each group were anesthetized (i.p.) with 10% chloral hydrate, blood samples were taken from the abdominal aorta and collected in heparin-sodium tubes, and intestinal lavage fluid was collected by instilling the intestines with 30% ethanol. Plasma and intestinal lavage fluid samples were collected by centrifugation (4°C, 5,702 g, 5 min) and stored at -80°C prior to analysis.

### 16S rRNA Microbial Community Analysis

The differences in the gut microbial communities between the Control and Model groups were determined by collecting fecal samples from 10 randomly selected rats in each group for 16S rRNA gene analysis. Total DNA was extracted using the E.Z.N.A. Stool DNA kit (Omega Bio-Tek, Norcross, GA, USA).

The V3–V4 region of the bacterial 16S rRNA gene was polymerase chain reaction (PCR)-amplified using the following primers: 338F 5’ -ACTCCTACGGGAGGCAGCA-3’ and 806R 5’- GGACTACHVGGGT WTC TAAT-3’. PCR was performed under the following conditions: 95°C for 3 min, followed by 27 cycles of 95°C for 30 s, 55°C for 30 s, and 72°C for 45 s, with a final step at 72°C for 10 min. Amplification was confirmed by 2% agarose gel electrophoresis. PCR products were purified with the AxyPrep DNA kit (AXYGEN, Tewksbury, MA, USA) and sequenced on the Illumina HiSeq platform. The PE reads obtained by MiSeq sequencing were first spliced according to the overlap relationship, and the quality of the sequences was controlled and filtered simultaneously. After distinguishing the samples, community bar plot analysis was performed at the phylum and genus levels. β-Diversity was estimated by the Bray–Curtis distance algorithm and visualized by principal coordinate analysis (PCoA). The Phylogenetic Investigation of Communities by Reconstruction of Unobserved States (PICRUSt) was used to predict function and obtain COG family information corresponding to Operational Taxonomic Units (OUT). The description and function of each COG were searched in the eggNOG database to obtain a functional abundance spectrum. The data were analyzed on the free online Majorbio I-Sanger Cloud Platform (www.i-sanger.com).

### Plasma and Intestinal Lavage Fluid Metabolomics

#### Sample Preparation

A working internal standard (IS) solution of 2-chloro-l-phenylalanine (10 µg/mL) was prepared in methanol. Plasma samples (50 µL) were added into 200 µL working IS solution. The mixture was vortexed for 3 min and centrifuged (17,108 g, 15 min, 4°C). After centrifugation, the supernatant was collected and stored at -20°C. Intestinal lavage fluid samples (5 mL) were condensed to half the original volume by nitrogen flow. Concentrated samples (200 µL) were almost completely dried under nitrogen flow, then re-dissolved in IS solution (200 µL), vortexed and centrifuged (17,108 g, 15 min, 4°C). The collected supernatant was analyzed by UPLC-Q-TOF/MS. Furthermore, significant differences between groups were analyzed based on community abundance data.

#### UPLC-Q-TOF/MS Analysis

Chromatographic analysis was performed on the Waters ACQUITY UPLC System (Waters Corp. Milford, USA). The samples were separated in an Acquity UPLC HSS T3 column (100 mm × 2.1 mm, 1.8 µm) at 40°C and a flow rate of 0.35 mL/min with a mobile phase composed of 0.1% formic acid water (A) and acetonitrile (B). The gradient program for plasm samples was optimized as follows: 0–0.2 min, 5% B; 0.2–3 min, 5% B to 20% B; 3–5 min, 20% B to 45% B; 5–7 min, 45% B to 55% B; 7–13 min, 55% B to 65% B; 13–16min, 55% B to 65% B; 16–21min, 65% B to 80% B; 21–23min, 80% B to 95% B; and 23–26 min, equilibration with 5% B. The gradient program for intestinal lavage fluid samples was optimized as follows: 0–0.5 min, 1% B; 0.5–3 min, 1% B to 30% B; 3–8 min, 30% B to 40% B; 8–20 min, 40% B to 60% B; 20–22 min, 60% B to 99%, 22–23 min, washing with 99% B, and 23–26 min, equilibration with 1% B. Mass spectrometry analysis was performed using the Waters SYNAPT G2 system with an ESI source operating in both positive and negative ion modes. Briefly, the parameters of the positive ion mode were set as follows: capillary voltage, 4.0 kV; source temperature, 120°C; cone gas rate, 40 L/h; desolvation gas rate and temperature, 800 L/h and 400°C; cone voltage, 19 V; collision energy 20–40 V; scan time and inter-scan delay, 0.15 and 0.02 s. The parameters used in the negative ion mode were the same as those used in the positive ion mode except: capillary voltage, 2.5 kV; cone voltage, 25 V. For quality calibration, leucine-enkephalin (0.5 µg/mL) was used as the lockmass in all analyses ([M+H]^+^ = 556.2771, [M-H]^-^ = 554.2615) with a flow rate of 5 µL/min. Profile data were collected in MSE mode from m/z 50 to 1,250 Da.

#### Method Validation

To ensure the stability and repeatability of our sequence analysis, equal volumes (10 µL) of each plasma sample and intestinal lavage fluid sample were pooled as quality control (QC) samples and then prepared in the same way as the test samples. The QC sample was injected every 10 samples during the batch analysis. Subsequently, 10 typical peaks (including the IS peak) were extracted for method validation. The relative standard deviation (R.S.D.%) values of the intensity are shown in **Tables S1**, **S2**, and the base peak intensity (BPI) chromatograms of the QC samples showed good concordance (**Figures S2** and **S3**).

#### Data Processing and Multivariate Analysis

All of the raw LC-MS files of the Control and Model groups were imported to Progenesis QI V 2.0 software for data processing. The converted files were subjected to alignment, peak-picking, and deconvolution. The filtered data were exported to EZinfo 3.0 for principal component analysis (PCA) and orthogonal partial least square discriminate analysis (OPLS-DA). Combined VIP-plots (VIP >1) were generated from the OPLS analysis to select distinct variables as potential markers. Tags (VIP >1 and *P* < 0.05) were established to screen and identify differentially expressed compounds. ChemSpider (http://www.chemspider.com/), the Human Metabolome Database (HMDB, http://www.hmdb.ca/), and the Kyoto Encyclopedia of Genes and Genomes (KEGG, http://www.kegg.jp/) database were selected for metabolite identification based on a combination of database queries using the exact mass measurements (mass error <5 ppm) and MS/MS patterns measured by the Q-TOF platform. Other parameter settings were designated as default for automatic data processing.

### Thermal Sensitivity Test

Thermal sensitivity tests were performed at 1, 3, 5, 7, and 9 days between 10:00 a.m. and 12:00 p.m. in a quiet room using the ZH-200 thermal stabbing pain instrument (Anhui Zhenghua Instrument Equipment Co., Ltd.). All animals were acclimatized to the test room. After the rats stop moving, the right hind paw of rats was stimulated by infrared radiation stimulated with a cut-off time for 15 s to prevent burns. When the rats lifted or licked the hind foot, the light was switched off manually, and the duration of the stimulus was recorded. This procedure was repeated three times (at 5-min intervals) for each rat, and the average value was calculated as the photorespiration threshold time.

### Determination of Plasma Biochemical Indicators

The plasma concentrations of calcitonin gene-related peptide (CGRP), *endothelin* 1 (ET 1), serotonin (5-HT), and nitric oxide (NO) were determined using a rat-specific enzyme-linked immunosorbent assay (ELISA) kit and NO assay kit (Nanjing Jiancheng Bioengineering Institute, Nanjing, Jiangsu, China) according to the manufacturer’s instructions. All samples were tested in duplicate.

### Co-Incubation of Feces and GUW *In Vitro*

To verify that the *in vivo* effects of GUW on metabolism are mediated *via* intestinal bacteria, we collected feces from rats in the Control and Model groups. Samples were divided into four groups (control group (CG), model group (MG), model+GUW group (MGG), and model +GUW+ antibiotic group (MGA); n = 4 samples per group). Stool samples were weighed and added to Eppendorf (EP) tubes. Three volumes of water were added to the feces samples of CG and MG. GUW (2 mg/mL) was added to the MGG samples. GUW (2 mg/mL) and antibiotics (Metronidazole, 2 mg/L, Gentamicin Sulfate, 4 mg/L, and Ampicillin, 1 mg/L) were added to fecal samples of MGA. The samples were placed in a strong plastic anaerobic box, and an anaerobic gas bag was placed in the box to simulate an anaerobic environment. The box was then placed in a Constant Temperature Foster Box (*Sanyo* Electric Co., Ltd., *Japan*). The samples were incubated in the anaerobic box at 37°C for 24 h. They were then centrifuged (7,128 g, 5 min, 4°C), and the supernatants were collected and stored at -20°C.

### Statistical Analysis

Statistical analysis was performed using IBM SPSS Statistics 21.0 (Chicago, USA). Differences between groups were evaluated by one-way analysis of variance (ANOVA). Multi-omics data were analyzed by Pearson’s correlation analysis. The significance threshold was set at *P* < 0.05 for all tests.

## Results

### Identification of Chemical Constituents in Extracts of *Gastrodia-Uncaria*

**Figure 1:**
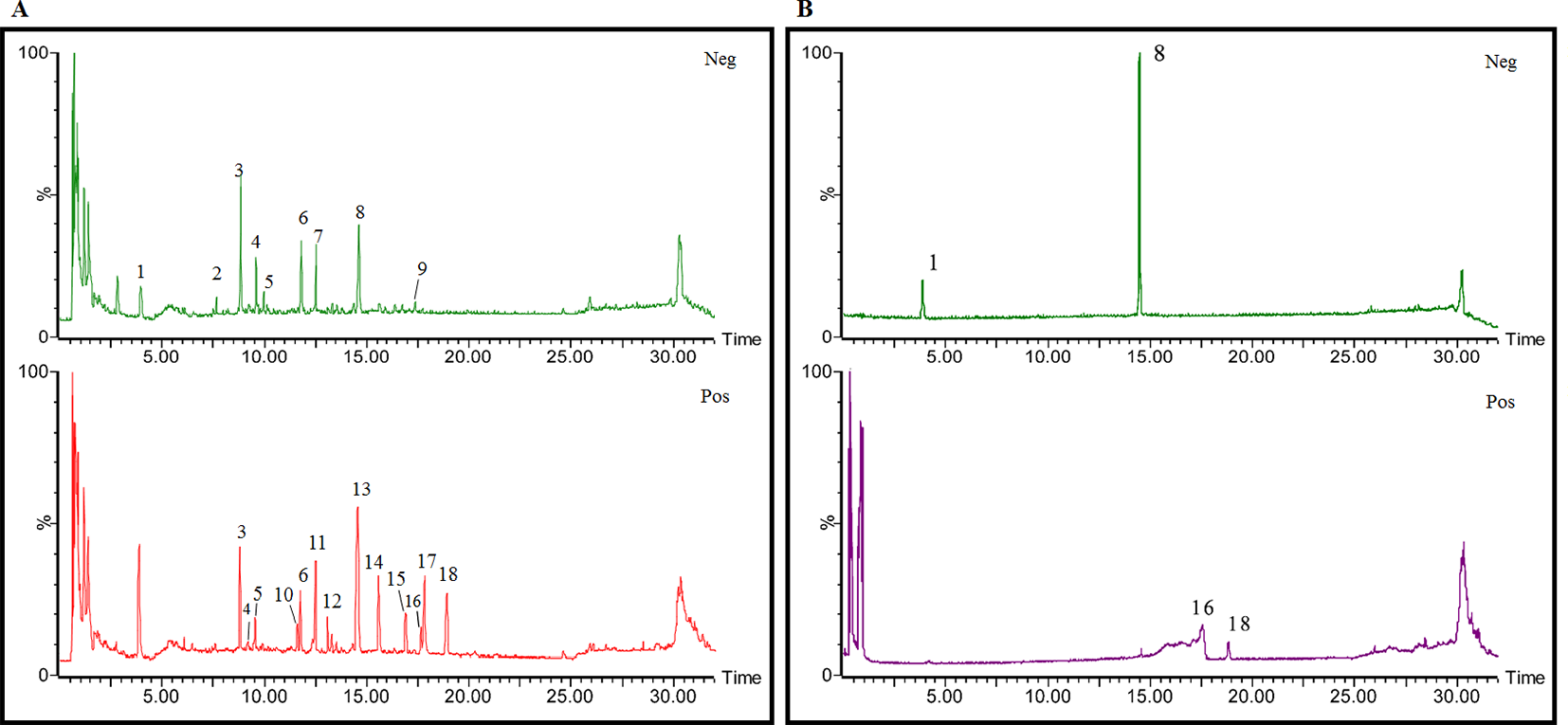
UPLC-Q-TOF chromatograms of GU extraction and Standers **(A)**; Standers **(B)**.

The total ion chromatogram (TIC) profiles of the compounds are shown in **Figure 1**, and detailed information on these chemical compounds is given in **Table 1**. Eighteen compounds were identified in the GU extract, of which four major active ingredients, gastrodin, parishin A, rhynchophylline, and isorhynchophylline, were identified using standards (Herbpurify Co., Ltd, Chengdu, China) and others were identified by examining their characteristic product ions. Peak 8 was identified as Parishin A by comparison with the standard and observing its fragment ions at m/z727.2248, 441.1115, and 423.0990. Similar to Peak 8, Peaks 3, 6, and 7 were identified as Parishin E, Parishin B, and Parishin C, respectively (Li et al., 2015). Peak 14 presented a characteristic product ion [M+H]^+^ at m/z 385.2134. It was identified as rhynchophylline by using standards, and the main fragment ions included m/z353.1861, 267.1454, 241.1368, 187.0890, and 160.0741. The characteristic ions of Peaks 12, 14, 15, 17, and 18 were similar to Peak 14, and these peaks were identified as demethyl rhynchophylline, demethyl isorhynchophylline, dehydrogen rhynchophylline, dehydrogen isorhynchophylline, and isorhynchophylline (Pan et al., 2017).

**Table 1:** Identification of the main components in GU extract based on UPLC-Q-TOF-MS.

NO	Name	Formula	t_R_	Mass Observed	Mass Errol (ppm)	Fragment ions	Structure
1	Gastrodin	C_13_H_18_O_7_	3.87	331.1020 [M+COOH]^-^	-0.3	123.0440	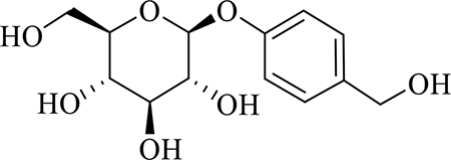
2	5-caffeoylquinic acid	C_16_H_18_O_9_	7.63	353.0844 [M-H]^-^	-4.5	191.0593, 179.0350, 173.0487, 135.0433	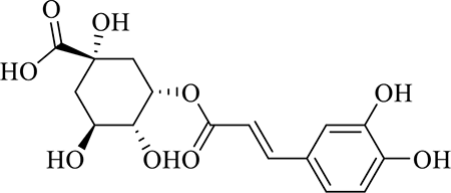
3	Parishin E	C_19_H_24_O_13_	8.84	459.1150 [M-H]^-^	3.7	173.0085, 190.9929, 123.0440	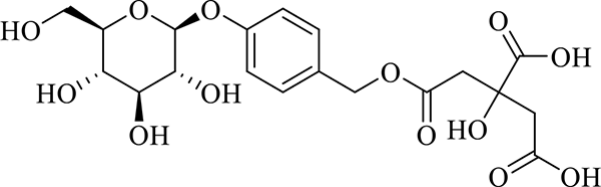
4	3-caffeoylquinic acid	C_16_H_18_O_9_	9.58	353.0844 [M-H]^-^	-4.5	191.0593, 179.0350, 173.0487, 161.0266	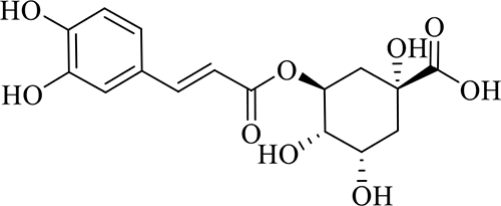
5	4-caffeoylquinic acid	C_16_H_18_O_9_	9.95	353.0844 [M-H]^-^	-4.5	191.0593, 179.0350, 173.0487, 161.0266, 135.0484	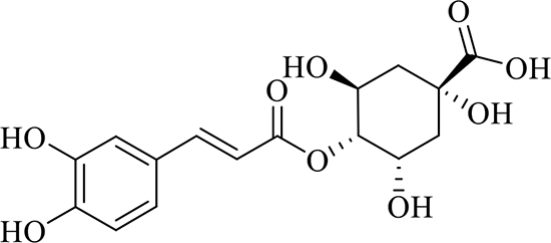
6	Parishin B	C_32_H_40_O_19_	11.80	727.2087 [M-H]^-^	0.6	441.1115, 423.0990, 161.0487	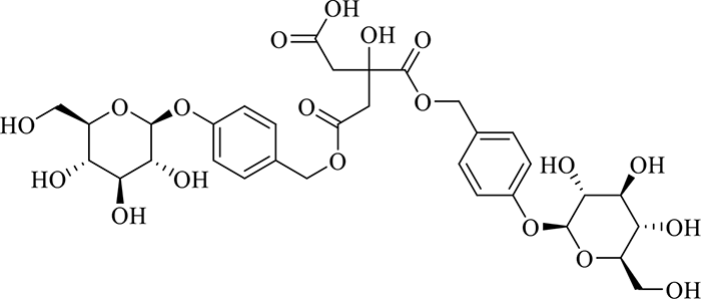
7	Parishin C	C_32_H_40_O_19_	12.48	727.2087 [M-H]^-^	0.6	441.1115, 423.0990, 161.0487	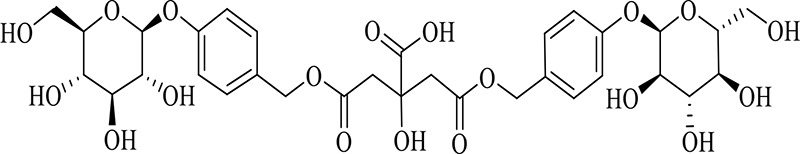
8	Parishin A	C_25_H_56_O_25_	14.58	995.3038 [M-H]^-^	0.7	727.2248, 441.1115, 423.0990	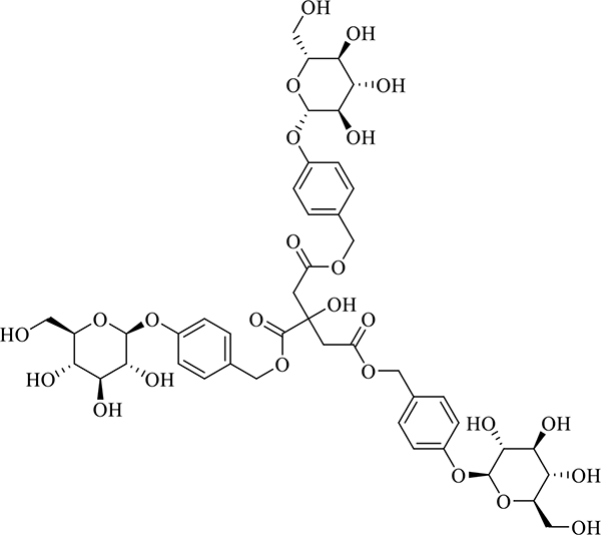
9	4,5-dicaffeoylquinic acid	C_25_H_24_O_12_	17.34	515.1204 [M-H]^-^	1.3	353.0944, 335.0769, 191.0593, 179.0350, 173.0487, 135.0484	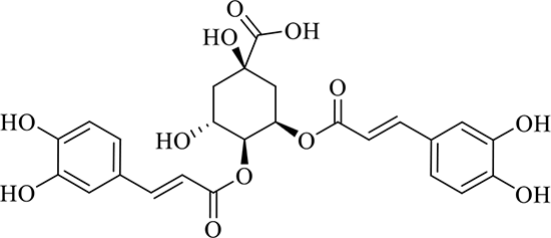
10	Strictosidine	C_27_H_34_N_2_O_9_	11.58	531.2352 [M+H]^+^	1.9	369.1826, 301.1386, 267.1454, 193.0497, 160.0741	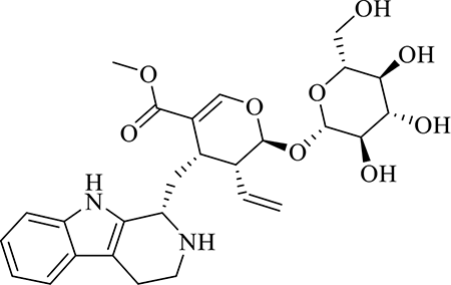
11	uncarine A	C_21_H_24_N_2_O_4_	12.45	369.1826 [M+H]^+^	3.6	351.1706, 267.1525, 160.0741	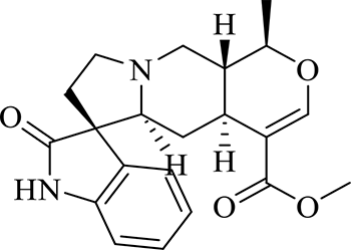
12	Demethyl Rhynchophylline	C_21_H_26_N_2_O_4_	13.07	371.1978 [M+H]^+^	2.3	267.1454, 241.1368, 187.0890, 160.0741	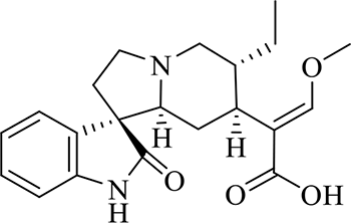
13	uncarine C	C_21_H_24_N_2_O_4_	14.55	369.1826 [M+H]^+^	3.6	367.1525, 192.1027, 160.0741	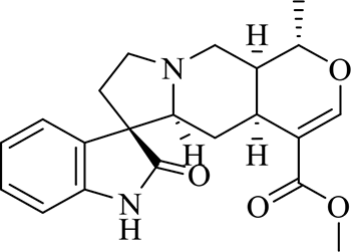
14	Demethyl Isorhynchophylline	C_21_H_26_N_2_O_4_	15.57	371.1978 [M+H]^+^	2.3	269.1616, 353.1861, 241.1368, 160.0741	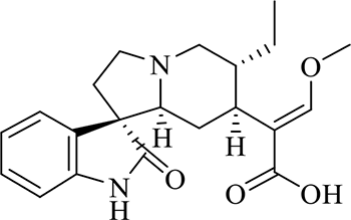
15	Dehydrogen Rhynchophylline	C_22_H_26_N_2_O_4_	16.87	383.1967 [M+H]^+^	-1.1	351.1706, 267.1454, 187.0890, 160.0741	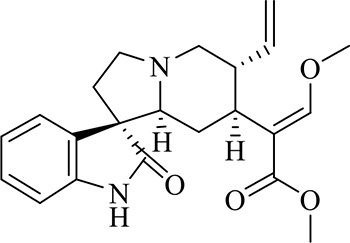
16	Rhynchophylline	C_22_H_28_N_2_O_4_	17.70	385.2134 [M+H]^+^	2.5	353.1861, 267.1454, 241.1368, 187.0890, 160.0741	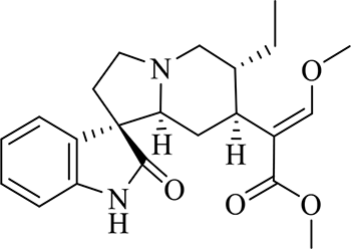
17	Dehydrogen Isorhynchophylline	C_22_H_26_N_2_O_4_	17.79	383.2053 [M+H]^+^	2.1	351.1706, 267.1525, 160.0741	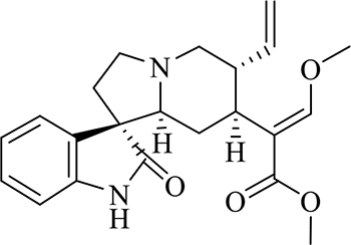
18	Isorhynchophylline	C_22_H_28_N_2_O_4_	18.87	385.2134 [M+H]^+^	2.5	353.1861, 269.1616, 241.1300, 160.0741	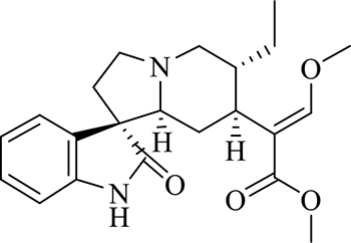

### Gut Microbiota Disorder in NTG-Induced CMG

**Figure 2:**
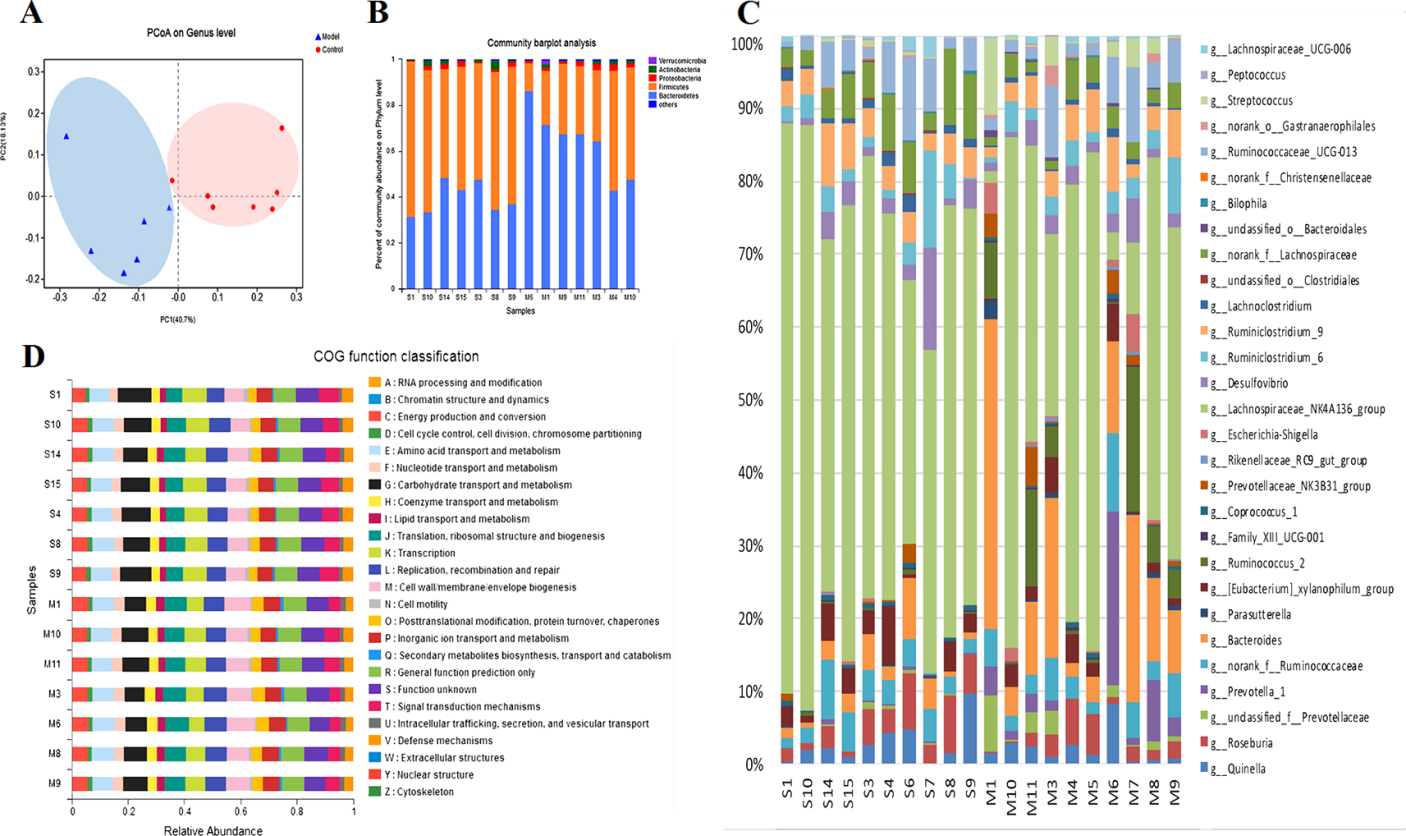
**(A)** The gut microbiota patterns of Control group and Model group differentiated by PCoA. The gut microbiota composition profiles at the phylum **(B)** and genus **(C)** levels in the Control and Model group as revealed by 16S rRNA gene sequencing; each color represents one bacterial phylum or genus. The x-axis represents different samples. The y-axis represents the percentage abundance of bacteria in each sample. **(D)** Abundances of PICRUSt-inferred function in the Control and Model samples.

The principal co-ordinate analysis (PCoA) of the Bray–Curtis genus horizontal distance showed significant separation between the Control and Model groups (**Figure 2A**); the first principal coordinate explained 40.7% of the variation, and the second principal coordinate explained 18.13% of the variation. Identification at the phylum level showed that the proportion of *Bacteroidetes* and *Firmicutes* exceeded 90%. Compared to the Control group, the abundance of *Firmicutes* in the Model group decreased significantly (*P* < 0.05), whereas the abundance of *Bacteroides* increased (**Figure 2B**). In addition, comparisons of the Model and Control groups revealed statistically significant differences among the 30 genus-level bacteria (*P* < 0.05, Mann-Whitney U test) (**Figure 2C**). In migraine model rats, metagenomic data indicated increases in the relative abundance of *Prevotella*_1, *Prevotellaceae*_unclassified, *Ruminococcaceae*_norank, *Bacteroides*, *Parasutterella*, *Bacteroidales*_unclassified, Family_XIII_UCG-001, *Gastranaerophilales*_norank, *Lachnospiraceae*_UCG-006, *Ruminococcus*_2, *Prevotellaceae*_NK3B31_group, *Rikenellaceae*_RC9_gut_group, *Streptococcus*, *Escherichia-Shigella*, and decreases in the relative abundance of other differential intestinal bacteria, such as *Desulfovibrio*, *Ruminococcaceae*_UCG-013, *Coprococcus*_1, *Lachnospiraceae*_NK4A136_group, *Christensenellaceae*_norank, *Ruminiclostridium*_6, *Ruminiclostridium*_9, *Bilophila*, *Lachnospiraceae*_norank, *Lachnoclostridium*, *Clostridiales*_unclassified, and *Peptococcus*.

Prediction of function using the Phylogenetic Investigation of Communities by Reconstruction of Unobserved States (PICRUSt) indicated that the main changes occurred in the amino metabolism, energy metabolism, and lipid metabolism pathways. The clusters of orthologous groups (COG) function classification is shown in **Figure 2D**. Compared with the Control group, the abundance of defense mechanisms, energy production and conversion, and amino acid transport and metabolism were decreased in the CMG group.

### Plasma Metabolic Profile of the Rat CMG Model and Identification of Potential Metabolite Biomarkers

**Figure 3:**
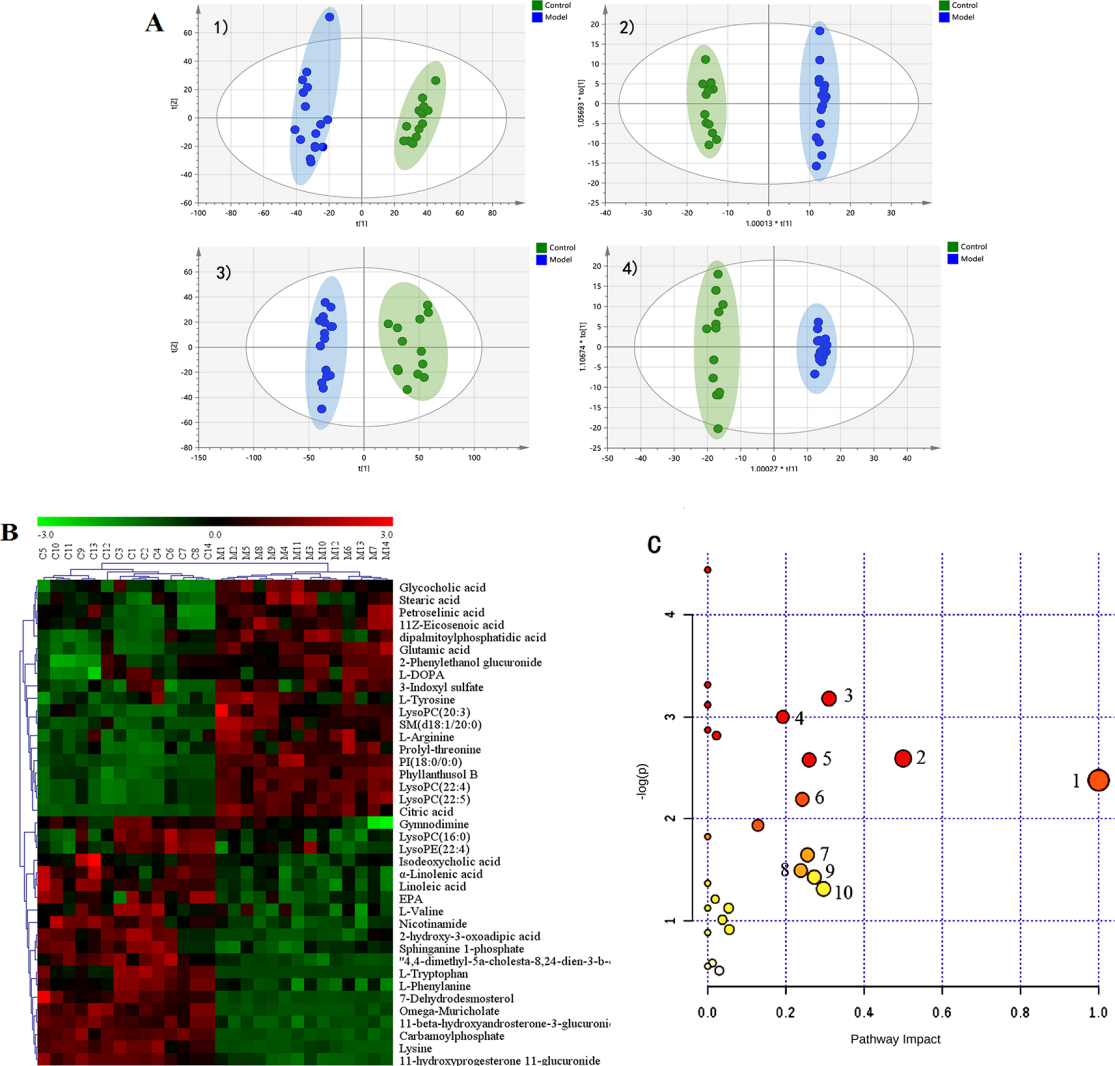
Metabolic profiles of plasma samples in rats with chronic migraine induced by nitroglycerin **(A)** 1) PCA score plot in negative mode; 2) OPLS-DA score plot in negative mode (R2Y = 0.997, Q2Y = 0.98); 3) PCA score plot in positive mode; 4) OPLS-DA score plot in positive mode (R2Y = 0.995, Q2Y = 0.973). **(B)** Significant changes in plasma metabolites are expressed as a heatmap showing metabolite changes in the Control (C) and Model (M) treatment groups, detected by UPLC-Q-TOF/MS. **(C)** Overview of metabolic pathway analysis: 1. Linoleic acid metabolism 2. Phenylalanine, tyrosine, and tryptophan biosynthesis 3. Tryptophan metabolism 4. Arginine and proline metabolism 5. Alanine, aspartate, and glutamate metabolism 6. Glycerophospholipid metabolism 7. Tyrosine metabolism 8. Nicotinate and nicotinamide metabolism 9. Pentose and glucuronate interconversions 10. Glyoxylate and dicarboxylate metabolism.

The metabolic profiles of plasma samples in each group were analyzed by UPLC-Q-TOF-MS/MS in both the positive and negative ion modes. All the groups tested were discriminated in the principal component analysis (PCA) model and orthogonal partial least square discriminate analysis (OPLS-DA), and a separation between the CMG and Control groups was clearly shown both in the PCA score plot and the OPLS-DA analysis (**Figure 3A**). Furthermore, the parameters obtained in the permutation of 200 tests indicated that the OPLS-DA models were reliable, with good predictability (**Figure S4**).

The OPLS-DA method was used to refine the separation between the CMG and Control groups already established *via* PCA. Dissimilar metabolites (VIP >1) were then imported into the QI program and tagged as contributive variables. Those filtered metabolites were selected to create a new quick tag (ANOVA *P* < 0.05). Metabolites that satisfied the parameters of VIP >1 and *P* < 0.05 were identified using the online databases (e.g., HMDB, ChemSpider, KEGG) with Progenesis QI, and the identification results were then exported. The accurate mass and MSE spectral measurements of the identification results, collected by UHPLC-Q-TOF/MS, were then matched with standards (**Figure S5**), and the identification was confirmed using the UNIFI Scientific Information System. In comparisons of the CMG Model and Control groups, 14 metabolites showed significantly different levels in the positive ion mode and 26 in the negative ion mode (**Table 2**). Differentially expressed biomarkers mainly consisted of indoles, carbohydrates, amines, organic acids, amino acids, neurotransmitters, bile acids, free fatty acids, and lipids. Variation in the identified plasma biomarkers related to NTG-induced CMG is depicted in the heatmap for each treatment group (**Figure 3B**). All 40 potential biomarkers were subjected to metabolic pathway analysis (MetPA) using the KEGG online database and MetaboAnalyst 3.0 (Xia and Wishart, 2010). An overview of the pathway analysis shown in **Figure 3C** reflects the metabolic network associated with the CMG model. For the rat plasma samples, the metabolic pathways were identified as linoleic acid metabolism, phenylalanine, tyrosine and tryptophan biosynthesis, tryptophan metabolism, arginine and proline metabolism, alanine, aspartate and glutamate metabolism, glycerophospholipid metabolism, tyrosine metabolism, nicotinate and nicotinamide metabolism, pentose and glucuronate interconversions, and glyoxylate and dicarboxylate metabolism.

**Table 2:** Identification of potential biomarkers of rat plasma samples between control group and chronic migraine module group.

NO	Retention Time	Name	Formula	Experimental Mass	Ion Mode	Mass Error	MS/MS
P1	7.12	omega-Muricholate	C24H40O5	407.2803	[M-H]-	1.7	389.2695, 346.2837, 289.2176, 209.1544, 195.1758
P2	11.44	Gymnodimine	C32H45NO4	506.3276	[M-H]-	-4.3	488.3161
P3	3.81	2-hydroxy-3-oxoadipic acid	C6H8O6	175.0248	[M-H]-	-1.1	157.0135, 129.0194,113.0245, 85.0297, 59.0142
P4	9.38	Glycocholic acid	C26H43NO6	500.2784	[M+Cl]-	-0.6	464.3020, 402.3008
P5	19.47	Petroselinic acid	C18H34O2	281.2486	[M-H]-	-0.7	281.2486, 263.2380
P6	21.27	Stearic acid	C18H36O2	283.2637	[M-H]-	-2.0	269.2487, 255.2329, 241.2180, 227.2017
P7	21.51	11Z-Eicosenoic acid	C20H38O2	309.2799	[M-H]-	-0.6	265.2895
P8	9.34	Isodeoxycholic acid	C24H40O4	391.2854	[M-H]-	-0.5	373.2738, 329.2844
P9	8.58	sphinganine 1-phosphate	C18H40NO5P	380.2571	[M-H]-	-1.1	253.2170, 224.0692, 163.1127
P10	2.65	*L*-Tryptophan	C11H12N2O2	203.0826	[M-H]-	-1.5	186.0568, 130.0666, 116.0510, 74.0254
P11	0.58	Lysine	C6H14N2O2	145.0982	[M-H]-	-0.7	131.0826, 113.0732
P12	0.73	glutamic acid	C5H9NO4	146.0465	[M-H]-	-1.6	128.0355, 102.0546
P13	0.62	*L*-Arginine	C6H14N4O2	173.1044	[M-H]-	-0.1	131.0826, 156.0732, 68.9956
P14	8.34	LysoPC(14:0)	C22H46NO7P	512.2994	[M+HCOO]-	-0.8	452.2784, 227.2017, 168.0430, 78.9592
P15	12.12	LysoPC(16:0)	C24H50NO7P	494.3252	[M-H]-	-0.8	462.2978, 421.2724, 269.2484, 224.0689, 78.9593
P16	9.81	LysoPE(22:4)	C27H48NO7P	528.309 6	[M-H]-	-0.8	483.2499, 457.2362, 303.2331, 259.2429,
P17	3.35	3-Indoxyl sulfate	C8H7NO4S	212.0023	[M-H]-	-1.9	132.0454, 80.9655
P18	4.83	2-Phenylethanol glucuronide	C14H18O7	297.098	[M-H]-	-0.7	204.0664, 175.0243, 121.0661
P19	9.49	Phyllanthusol B	C35H49NO18	816.2932	[M+HCOO]-	-1.5	628.2337, 507.2115, 346.1273, 168.0429
P20	10.33	LysoPC(20:3)	C28H52NO7P	590.3463	[M+HCOO]-	-1.4	530.3249, 460.2833, 305.2486
P21	11.99	LysoPC(22:4)	C30H54NO7P	616.362	[M+HCOO]-	0.2	556.3398, 331.2634
P22	14.11	PI(18:0/0:0)	C27H53O12P	599.3202	[M-H]-	0.3	419.2563, 315.0487, 241.0117, 152.8859, 78.9592
P23	17.62	dipalmitoylphosphatidic acid	C35H69O8P	629.4552	[M-H2O-H]-	-0.2	629.4542
P24	11.19	LysoPC(22:5)	C30H52NO7P	614.3463	[M+HCOO]-	-2.8	554.3245, 497.2881, 329.2483, 285.2598
P25	7.12	SM(d18:1/20:0)	C43H87N2O6P	837.5491	[M+Br]-	1.0	429.2628, 407.2809, 251.2020, 195.1758
P26	3.48	*L*-DOPA	C9H11NO4	178.051	[M-H2O-H]-	-1.1	178.0507, 134.0613
P27	2.65	*L*-Tryptophan	C11H12N2O2	205.0972	[M+H]+	-3.5	159.0952, 144.0831, 118.0683, 90.0482, 75.0249
P28	16.63	Linolenic acid	C18H30O2	279.2397	[M+H]+	-5.3	261.2286
P29	18.04	Linoleic acid	C18H32O2	263.2443	[M-H20+H]+	-0.6	263.2463, 121.1044
P30	1.94	*L*-Phenylalanine	C9H11NO2	166.0909	[M+H]+	-0.7	119.9480, 103.0005
P31	1.14	*L*-Tyrosine	C9H11NO3	182.0859	[M+H]+	-0.6	135.8930, 95.0005
P32	0.8	*L*-Valine	C5H11NO2	118.0862	[M+H]+	-1.1	118.0862
P33	1.07	Nicotinamide	C6H6N2O	123.0549	[M+H]+	-1.5	108.0439, 80.0490
P34	5.43	prolyl-threonine	C9H16N2O4	217.1261	[M+H]+	-0.7	102.0550
P35	20.17	11-beta-hydroxyandro-sterone-3-glucuronide	C25H38O9	483.2654	[M+H]+	-1.6	289.2164, 177.0396
P36	8.53	4,4-dimethyl-5a-cholesta-8,24-dien-3-b-ol	C29H50O	437.3815	[M+Na]+	0.0	463.2347, 203.1849, 133.1047, 105.0726
P37	1.05	Carbamoylphosphate	CH4NO5P	141.9642	[M+H]+	-0.8	98.9847
P38	1.96	Citric acid	C6H8O7	210.0590	[M+NH4]+	-0.8	193.0348, 133.0133
P39	11.43	7-Dehydrodesmosterol	C27H42O	400.3555	[M+NH4]+	-0.8	365.3203
P40	9.98	11-hydroxyprogesterone 3-glucuronide	C27H38O9	507.2661	[M+H]+	-1.9	331.2273, 177.0395

### Relevance Analysis Between Plasma Biomarkers and Gut Microbiota

**Figure 4:**
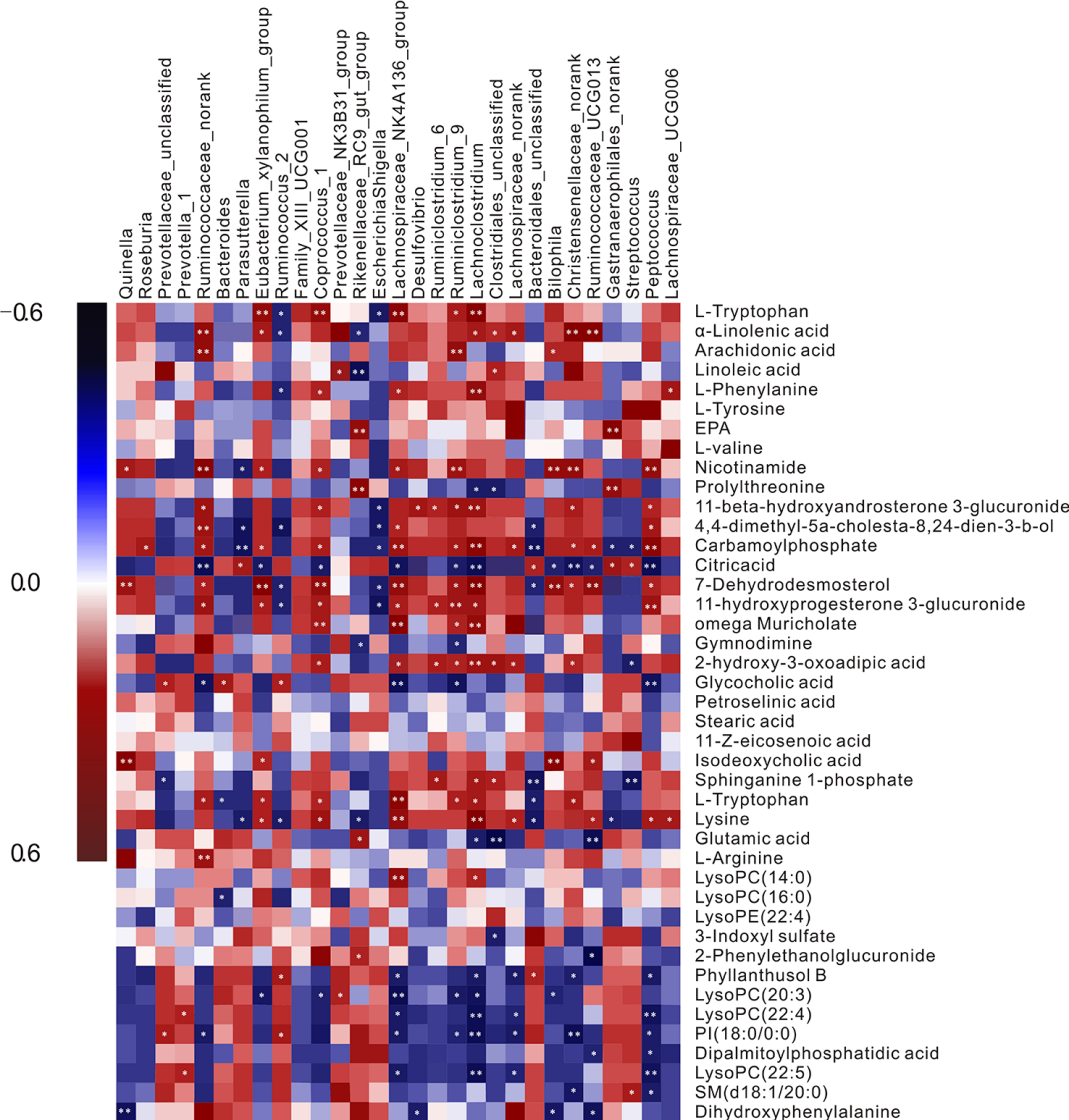
A correlation heatmap is used to represent significant statistical correlation values (*r*) between perturbed gut microbiota genera and altered plasma metabolites in the Model group and Control group. Blue squares indicate negative correlations, and red squares indicate positive correlations; the significance of correlation is closely related to the depth of color.

Pearson’s correlation analysis was performed to evaluate potential links between gut microbiota genera and plasma biomarkers (range for correlation, r >0.4 or r < −0.4; *P* < 0.05). In the correlation analysis, associations between the perturbed gut microbiota and altered plasma metabolites were identified. As was shown in **Figure 4** L-tryptophan (P10) was positively related to *Ruminiclostridium*_9, *Coprococcus*_1, *Lachnospiraceae*_NK4A136_group, and *Lachnoclostridium* but negatively related to *Bacteroides*, *Ruminococcus*, and *Escherichia-Shigella*. *L*-Arginine (P13) showed highly positive correlations with *Lachnoclostridium* and *Clostridiales*_unclassified, whereas glutamic acid (P12) had the opposite correlation with the two bacteria. *L*-Tyrosine (P31) correlated positively with *Prevotella*_1 and negatively with *Christensenellaceae*_norank. Significant negative correlations were discerned between *L*-DOPA (P26) and *Bilophila*. The intensity of 3-indoxyl sulfate (P17) was negatively correlated with the abundance of *Clostridiales*_unclassified. Nicotinamide (P33) was correlated positively with *Coprococcus*_1, *Bilophila*, *Ruminiclostridium*_9, and *Peptococcus*. These metabolites were involved in four key metabolic pathways, namely tryptophan metabolism (P10 and P17), arginine and proline metabolism (P12 and P13), nicotinate and nicotinamide metabolism (P33), and tyrosine metabolism (P31 and P26), and also represented a complete metabolome contributing to the formation of gut microbiota symbiosis.

### Intestinal Lavage Fluid Metabolic Profile of the Rat CMG Model and Identification of Potential Metabolite Biomarkers

**Figure 5:**
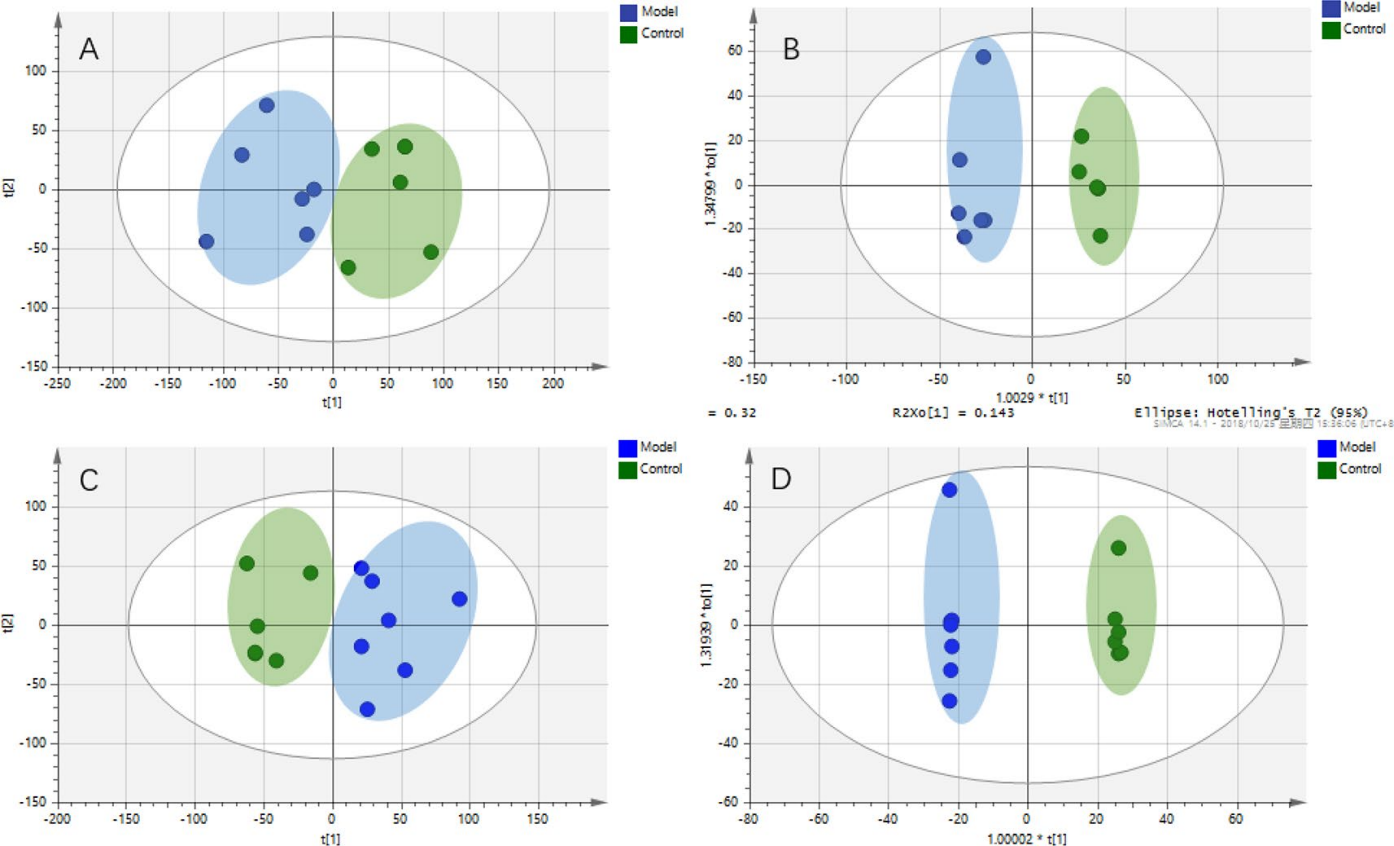
Metabolic profiles of intestinal lavage fluid samples in rats with chronic migraine induced by nitroglycerin. **(A)** PCA score plot in negative mode; **(B)** OPLS-DA score plot in negative mode (R^2^Y = 0.945, Q^2^Y = 0.748); **(C)** PCA score plot in positive mode; **(D)** OPLS-DA score plot in positive mode (R^2^Y = 0.999, Q^2^Y = 0.840).

Intestinal metabolites are directly influenced by gut microbial composition and affect plasma metabolomics by absorption into the blood (Windmueller and Spaeth, 1976); therefore, the correlation between intestinal flora and plasma metabolism may be caused by altered intestinal metabolism. Metabolic profiles of intestinal lavage fluid samples in the Control and Model groups were analyzed by UPLC-Q-TOF-MS/MS in both the positive and negative ion modes. The PCA and OPLS-DA (**Figure 5**) model indicated a complete separation between the metabolic profiles of rats in the two groups. The differential components of the intestinal lavage fluid were identified using the same methods used to identify the plasma biomarkers. In total, 12 metabolites were identified in intestinal lavage fluid, which is similar to the components of plasma metabolites identified (**Table 3**).

**Table 3:** Potential intestinal lavage fluid biomarkers in the rat model of chronic migraine induced by nitroglycerin.

NO	Retention Time	Name	Formula	Ion Mode	Error (ppm)	MS/MS
I1	11.03	omega-Muricholate	C24H40O5	[M-H]-	-0.12	389.2695, 289.2176, 209.1544, 195.1758
I2	0.88	Lysine	C6H14N2O2	[M-H]-	0.14	131.0826, 113.0732
I3	0.81	glutamic acid	C5H9NO4	[M-H]-	-0.07	128.0355, 102.0546
I4	0.74	*L*-Arginine	C6H14N4O2	[M-H]-	-0.12	131.0826, 156.0732, 68.9956
I5	3.54	3-Indoxyl sulfate	C8H7NO4S	[M-H]-	-0.14	132.0454, 80.9655
I6	11.03	SM(d18:1/20:0)	C43H87N2O6P	[M+Br]-	-0.20	429.2628, 407.2809, 251.2020, 195.1758
I7	3.59	dihydroxyphenylalanine	C9H11NO4	[M-H2O-H]-	0.11	178.0507, 134.0613
I8	3.04	*L*-Tryptophan	C11H12N2O2	[M+H]+	-1.12	159.0952, 144.0831, 118.0683, 90.0482, 75.0249
I9	2.65	*L*-Phenylalanine	C9H11NO2	[M+H]+	1.81	119.9480, 103.0005
I10	2.22	*L*-Tyrosine	C9H11NO3	[M+H]+	-0.77	135.8930, 95.0005
I11	0.83	*L*-Valine	C5H11NO2	[M+H]+	-0.85	118.0862, 78.0805
I12	15.69	11-beta-hydroxyan- drosterone-3-glucuronide	C25H38O9	[M+Na]+	2.47	289.2164, 177.0396

### Effect of GUW Treatment on Gut Microbiota Dysbiosis

**Figure 6:**
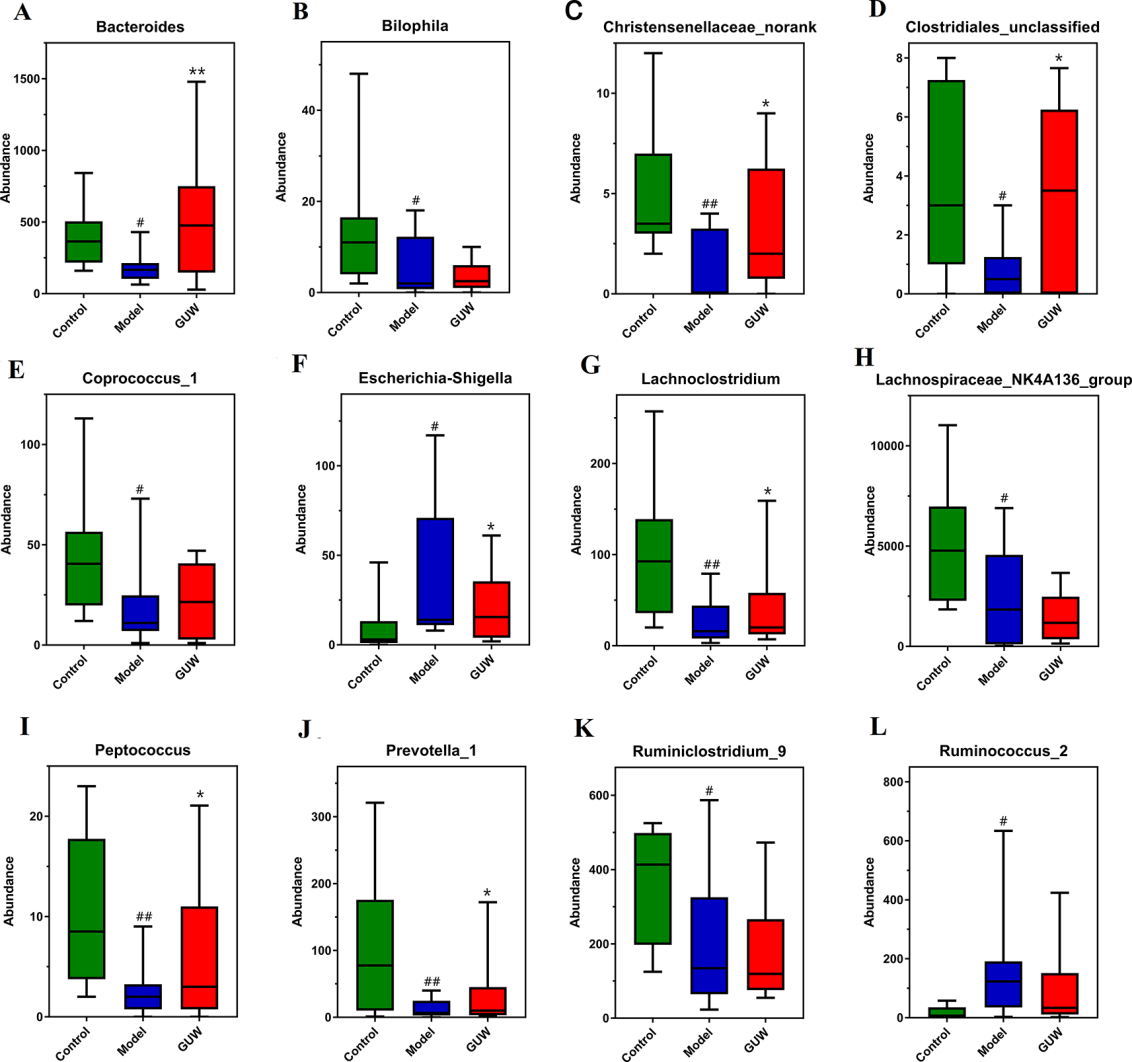
Regulation of GU on intestinal bacteria in the Model group, *P < 0.05, **P < 0.01 compared with the Model group; ^#^ *P* < 0.05, ^##^ *P* < 0.01 compared with the Control group. (Control, Control group; Model, Model group; GUW, GU administration of rats in the Model group.) **(A)** Bacteroides; **(B)** Bilophila; **(C)** Christensenellaceae_norank; **(D)** Clostridiales_unclassified; **(E)** Coprococcus_1; **(F)** Escherichia-Shigella; **(G)** Lachnoclostridium; **(H)** Lachnospiraceae_NK4A136_group; **(I)** Peptococcus; **(J)** Prevotella_1; **(K)** Ruminiclostridium_9; **(L)** Ruminococcus_2.

After GUW treatment, chronic NTG-induced dysregulation of bacterial abundance was improved at the genus level. Changes in the abundance of bacteria are shown in **Figure 6**. Compared with the Model group, the abundances of *Ruminococcus*_2 and *Escherichia-Shigella* were reduced in the GUW group. The abundances of *Bacteroides*, *Lachnoclostridium*, *Clostridiales*_unclassified, *Bilophila*, *Peptococcus*, and *Christensenellaceae*_norank were similar to those in the Control group. There were no obvious changes in the abundances of *Prevotella*_1, *Coprococcus*_1, *Ruminiclostridium*_9, and *Lachnospiraceae*_NK4A136_group.

### Effect of GUW Treatment on Plasma Metabolomics

To show the effect of GUW on the plasma metabolomics of the rat CMG model, the data for all groups (Control, Model, and CMG groups) were imported into SIMCA-P 14.1 to obtain a comprehensive metabolic profile. PCA and OPLS-DA analysis were performed to show the effect of GUW administration (**Figure S6**). The profiles of the three groups were obviously distinct, and the Model group was far from the Control group. The GUW group was between the Model group and the Control group in the x-axis direction, indicating differences between the groups. To avoid overfitting and random effects, a permutation of 200 tests was also performed to confirm that the OPLS-DA models were reliable with good predictability (**Figure S6**).

**Figure 7:**
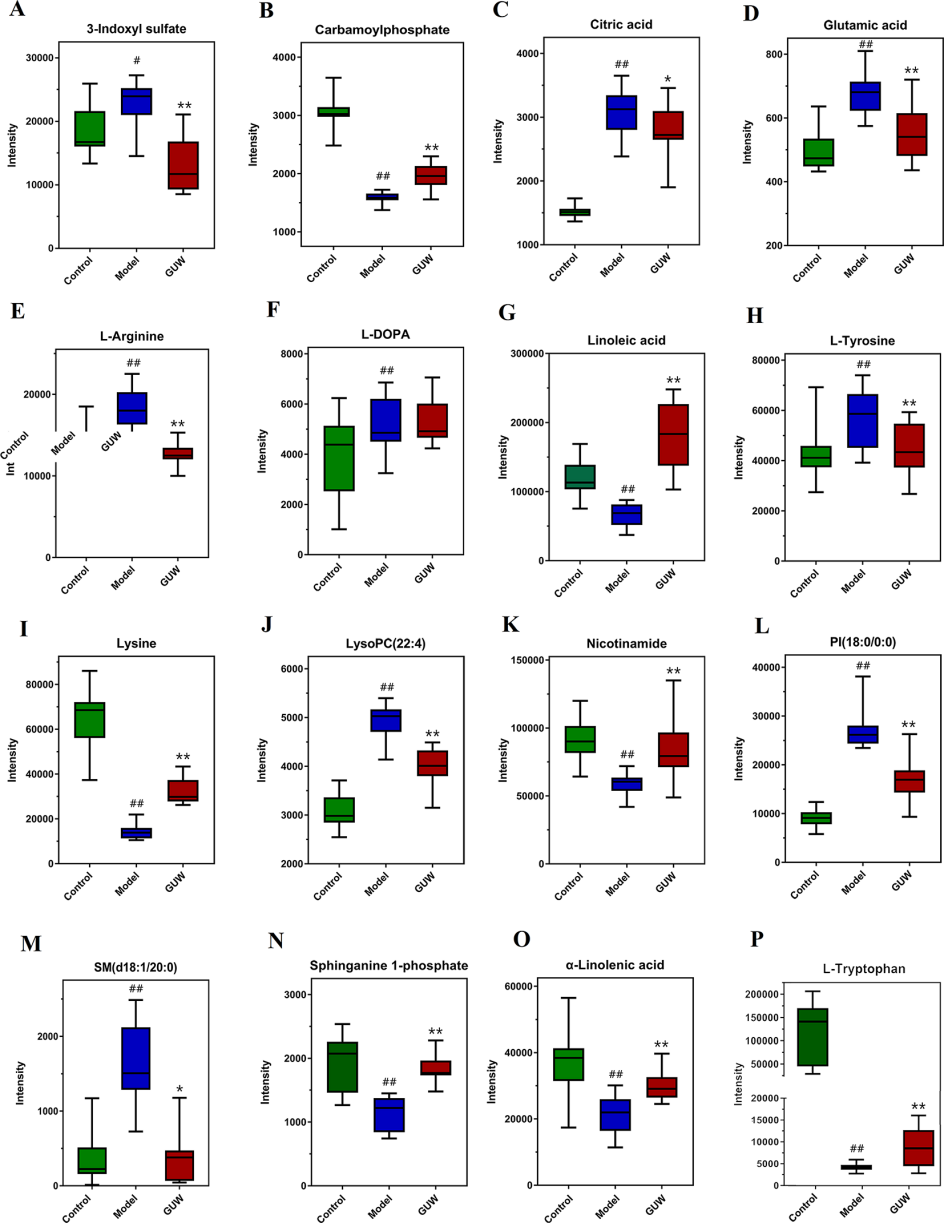
Expression of plasma biomarkers in all groups, *P < 0.05, **P < 0.01 compared with the Model group; ^#^ *P* < 0.05, ^##^ *P* < 0.01 compared with the Control group. (Control, Control group; Model, Model group; GUW, GU administration of rats in model group.) **(A)** 3-Indoxyl sulfate; **(B)** Carbamoylphosphate; **(C)** Citric acid; **(D)** Glutamic acid; **(E)** L-Arginine; **(F)** L-DOPA; **(G)** Linolenic acid; **(H)** L-Tyrosine; **(I)** Lysine; **(J)** LysoPC(22:4); **(K)** Nicotinamide; **(L)** PI(18:0/0:0); **(M)** SM(d18:1/20:0); **(N)** Sphinganine 1-phosphate; **(O)** α-Linolenic acid; **(P)** L-Tryptophan.

After GUW treatment, 28 endogenous metabolites that were disordered in CMG rats recovered to almost normal levels. The changes in 16 metabolites are listed in **Figure 7**, including *L*-tryptophan, *L*-arginine, glutamic acid, *L*-tyrosine, *L*-DOPA, 3-indoxyl sulfate, linoleic acid, and nicotinamide. These potential biomarkers are related to tryptophan metabolism, arginine and proline metabolism, nicotinate and nicotinamide metabolism, tyrosine metabolism, and linoleic acid metabolism. The other biomarkers are listed in **Figure S7**.

### Validation Study

#### The Effect of GUW Treatment Thermal Hyperalgesia Induced by Chronic NTG

**Figure 8:**
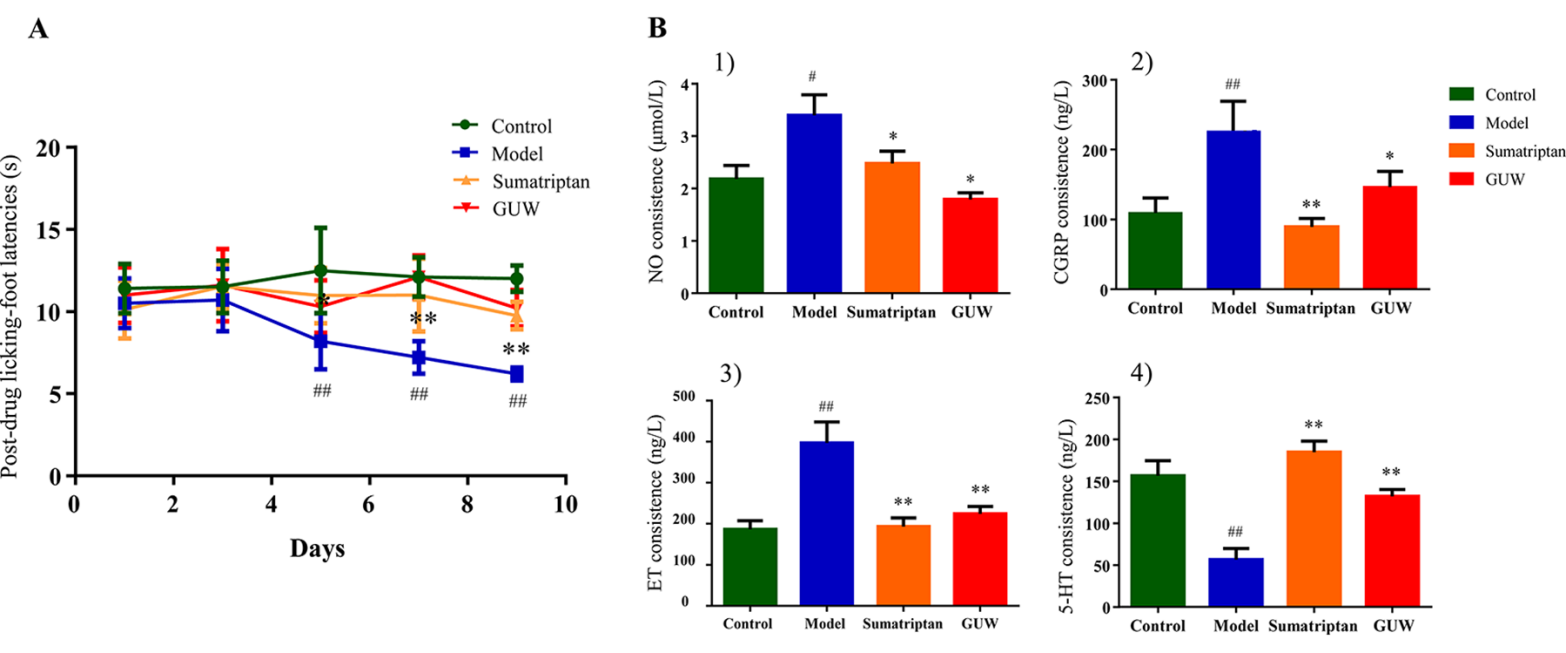
Chronic NTG-evoked hyperalgesia, which was significantly attenuated by GUW treatment. Data are expressed as mean ± SD (n = 10), *P < 0.05, **P < 0.01 compared with the Model group; ^#^ *P* < 0.05, ^##^ *P* < 0.01 compared with the Control group **(A)**. Regulation of plasma biochemical indicators levels induced by NTG administration and its reduction by GUW treatment. 1) NO, 2) CGRP, 3) ET, 4) 5-HT. Data are expressed as mean ± SD (n = 10), *P < 0.05, **P < 0.01 compared with the Model group; ^#^ *P* < 0.05, ^##^ *P* < 0 .01 compared with the Control group **(B)**.

Some studies have shown that CMG model rats exhibited thermal allodynia (Bates et al., 2010). Thermal sensitivity tests showed that NTG induced significant thermal hyperalgesia after the third day of treatment (on days 5, 7, and 9). Treatment with Sumatriptan and GUW for 10 days significantly increased thermal thresholds, and no significant differences were observed compared with the Control group (**Figure 8A**).

#### Effect of GUW Treatment on Plasma Concentration of 5-HT, CGRP, ET, and NO

Plasma biochemical indicators (5-HT, CGRP, ET, and NO) are closely related to chronic migraine models and are commonly used to indicate disease progression (Cui et al., 2017). Our study demonstrated that, compared with the Control group, chronic intermittent NTG administration increased plasma levels of NO (*P* < 0.05), CGRP (*P* < 0.01), and ET (*P* < 0.01) and reduced 5-HT levels (*P* < 0.01). After Sumatriptan and GUW treatment, there were no significant differences in plasma levels of NO, CGRP, ET, and 5-HT compared with those in the Control group (**Figure 8B**).

#### Co-Incubation of Feces and GUW *In Vitro*

**Figure 9:**
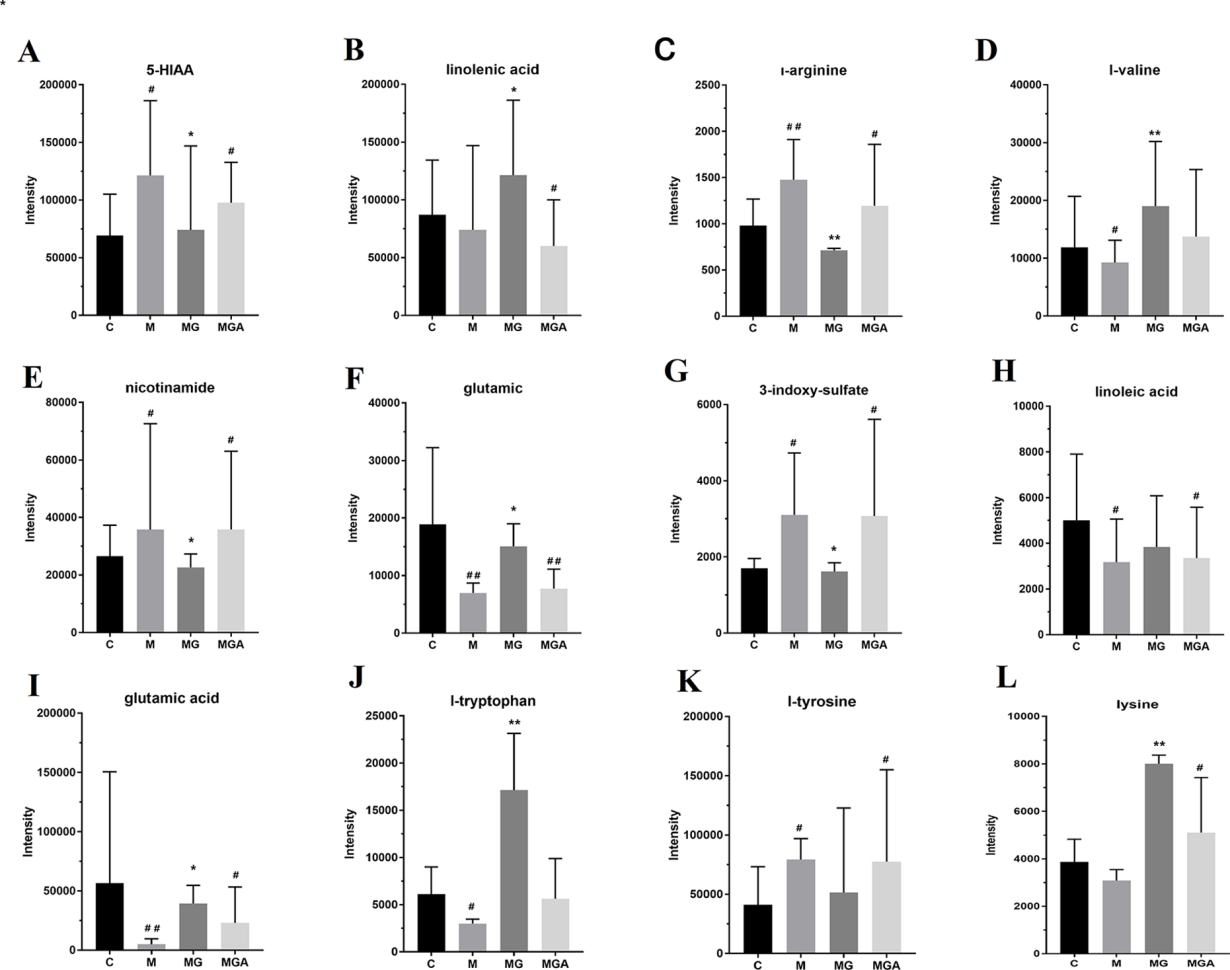
Effect of *in vitro* co-incubation of fecal samples on fecal bacterial metabolite levels, *P < 0.05, **P < 0.01 compared with the Model group; ^#^ *P* < 0.05, ^##^ *P* < 0.01 compared with the Control group. (C, feces of Control group; M, feces of Model group; MG, water extraction of GU added into feces of Model group; MGA, water extraction of GU and antibiotic mixed solution added into feces of Model group.) **(A)** 5-HIAA; **(B)** linolenic acid; **(C)** l-arginine; **(D)** l-valine; **(E)** nicotinamide; **(F)** glutamic; **(G)** 3-Indoxyl sulfate; **(H)** linoleic acid; **(I)** glutamic acid; **(J)** l-tryptophan; **(K)** l-tyrosine; **(L)** lysine.

To verify the *in vivo* effects of GUW on metabolism *via* intestinal bacteria, samples were divided into three groups (model group (MG), model+GUW group (MGG), and model +GUW+ antibiotic group (MGA); n = 4 samples per group) for short-term *in vitro* incubation. The samples were analyzed using UPLC-Q-TOF MS, and 19 components were identified in the feces samples that were associated with biomarkers in plasma. Since the metabolism of bacteria in the feces was inhibited after the addition of antibiotics, there were no significant differences in the metabolism of fecal bacteria in the MGA (*P* > 0.05) compared with MG (**Figure 9**). Levels of 3-indoxyl sulfate, glutamic acid, *L*-tyrosine, and *L*-arginine were reduced in MGG compared with in MG, and there were no significant differences between MG and MGA. Compared with MG, the levels of 5-HIAA, *L*-tryptophan, linoleic acid, and *L*-phenylalanine were increased after GUW treatment, and there were minimal changes in MGA.

## Discussion

Studying the response of microbial communities in the face of interference is critical to health (Costello et al., 2012), not only because the gut microbiota may change under disease conditions but also because the microbiota is affected by drugs administered to maintain host health (Zhang et al., 2019). It is increasingly recognized that the therapeutic effects of natural products on disease may be achieved through drug-microbial interactions, indicating the importance of research into the effects of drugs on microbial metabolism (Genda et al., 2018). In this study, we first determined the changes in the structure and function of the gut microbiota in a rat model of CMG. We found differences in the structure and function of the microbiota between the Model and Control groups. After GUW administration, dysregulation of the structure and function of the microbiota in the CMG rats was improved. We also observed that the significant changes in plasma metabolites in the Model group and drug-administered group varied with the changes in the intestinal microbiota.

Chronic NTG administration disturbed the microbial population, resulting in a significant decrease in the abundance of thick-walled bacteria and an increase in the relative abundance of *Bacteroides*. This result is consistent with the changes in the intestinal flora of autistic children (Finegold et al., 2010). In CMG model rats, metagenomic data indicated an increased relative abundance of *Prevotellaceae*_unclassified, *Prevotella*_1, *Ruminococcaceae*_norank, *Bacteroides*, *Parasutterella*, *Bacteroidales*_unclassified, Family_XIII_UCG-001, *Prevotellaceae*_NK3B31_group, *Ruminococcus*_2, *Gastranaerophilales*_norank, *Rikenellaceae*_RC9_gut_group, *Escherichia-Shigella*, and *Streptococcus*, while decreases were observed in the relative abundance of other differential intestinal bacteria, such as *Coprococcus*_1, *Lachnospiraceae*_NK4A136_group, *Ruminococcaceae*_UCG-013, *Desulfovibrio*, *Ruminiclostridium*_6, *Ruminiclostridium*_9, *Lachnoclostridium*, *Clostridiales*_unclassified, *Lachnospiraceae*_norank, *Lachnospiraceae*_UCG-006, *Bilophila*, *Christensenellaceae*_norank, and *Peptococcus*. It has been reported that women with high intestinal levels of *Prevotella*_1 show more negative emotions, such as anxiety and pain (Tillisch et al., 2017), which is consistent with our results. Lack of *Coprococcus* is found in most patients with depression, reflecting a link between *Coprococcus* and brain disease (Valles-Colomer et al., 2019). *Desulfovibrio* is associated with amino acid breakdown and ammonia production (Eschenlauer et al., 2002). The results of the present study indicate that the function of some bacterial strains may be related to chronic migraine; however, the function of most strains needs further verification.

Compared with the Control group, our metabolomics analysis showed differences in the levels of metabolites in the plasma and ileum of rats in the Model group. We found metabolites of interest in the Model group (*P* < 0.05, VIP >1 compared to the Control group). Previous metabolomics studies in this field have focused on transgenic migraine models and clinical patients (Shyti et al., 2015) and have shown disruption of biological metabolic pathways and changes in common metabolites, including free fatty acids and amino acids (Ren et al., 2018). Our study differs from previous studies in that we used a rat model of CMG induced by NTG for plasma metabolomics and intestinal lavage fluid analyses. Although model rats or clinical metabolomics are particularly relevant for the analysis of migraine biomarkers, exploring the potential correlation between plasma metabolites and endogenous markers in the ileum using this rat model of CMG is more indicative of the changes in the intestinal tract and the effect of changes in the flora on the overall metabolic level of the host. In addition, some intestinal strains have previously been shown to affect the progression of migraine disorders (Gonzalez et al., 2016). Analysis of differential markers of gut content allows us to focus on the differences in the compounds produced by changes in gut microbiota in the Model group and analyze their effects on host metabolism. *Clostridiales*_unclassified affects glutamate metabolism (Buckel, 2001), *Pseudomonas* affects plasma levels of nitrate (Gonzalez et al., 2016), *Coprococcus* seems to have a pathway associated with dopamine metabolism, and *Ruminococcus* metabolizes tryptophan to tryptamine (Williams et al., 2014). Although we have speculated that some strains are associated with plasma markers based on our data and earlier studies, whether these compounds can be regulated by the gut flora needs further verification.

GU is recognized as a natural medicine pair for the treatment of migraine. Currently, GU Granules (GUG) are marketed for the treatment of migraine. Studies have shown that few constituents of GUG migrate to the blood (Zhang et al., 2019), and some of the active ingredients, such as gastrodin, have a short half-life (Wu et al., 2017). Therefore, we consider that, in addition to the active ingredients into the blood, GU acts indirectly *via* another pathway. Intestinal flora analysis, a new method for studying the mechanism of action of natural medicines, was employed in our study. Using this approach, we validated the effect of GU in the rat model of CMG and determined the changes in the dysregulated flora and biomarkers in response to GU administration.

*In vitro* incubation of feces with antibiotics is the key to verifying whether the effects of GU are mediated *via* the intestinal flora. Intestinal bacteria are mostly anaerobic; therefore, we used an anaerobic box to simulate the internal environment. We did not perform these studies with a commonly used culture medium because these contain amino acids and other components that would interfere with the subsequent determination of endogenous components. Inhibition of the metabolism of intestinal bacteria by antibiotics showed that GU did not regulate the levels of components in the feces of the CMG model rats. In contrast, in the samples without antibiotics, the contents of some biomarkers were similar to those in the Control group after *in vitro* culture with GU, indicating that GU regulates the endogenous components in the intestine *via* the intestinal flora.

## Conclusion

The main contribution of this study is that clarification of the pathogenesis of migraine and the therapeutic effect of GUW is provided by the combination of 16S rRNA sequencing with metabolomics based on UPLC-MS. Our study shows that the therapeutic effect of GUW treatment on NTG-induced CMG rats is driven by the intestinal flora, which in turn regulates intestinal metabolism and the absorption of intestinal metabolites into the blood. After GUW administration, the abundance of disordered intestinal bacteria, including Ruminococcus_2, Escherichia-Shigella, Bacteroides, Lachnoclostridium, Bilophila, and Peptococcus, was adjusted, thereby affecting the metabolism of tryptophan, tyrosine, arginine, and niacin and nicotinamide to achieve therapeutic effects in NTG-induced CMG. Our findings provide new evidence of the role of the brain–gut axis in the mechanism by which food and natural medicines can be used to modulate the intestinal flora and achieve therapeutic effects in migraine. In future studies, we will conduct a flora transplantation experiment to confirm the role of characteristic intestinal flora in the treatment of migraine.

## Data Availability Statement

The datasets generated for this study can be found in the Sequence Read Archive, https://www.ncbi.nlm.nih.gov/sra/?term= SRP214751.

## Ethics Statement

The animal study was reviewed and approved by the Experimental Animal Ethics Committee of the State Key Laboratory (Reference number: BCTG-2016-18).

## Author Contributions

ZW wrote this main manuscript text and performed the data analysis. The animal experiment was conducted by ZW and CP together. In addition, MH, YR, and JL gave the contribution to plasma sample detection. As for data process, it is conducted by MH, ZL, and MZ. LD and YL contributed to manuscript preparation. HO designed the work that led to the submission, acquired data, and played an important role in interpreting the results. YF revised the manuscript. And SY contributed to the conception of the study.

## Funding

The study was funded by a grant (No. 81660651, 81560638) from National Natural Science Foundation of China, China Postdoctoral Science Foundation (No. 2017M612159), Postdoctoral Science Foundation of Jiangxi Province (No. 2017KY07), Natural Science Foundation of Jiangxi Province (No. 20171ACB21029, 20165BCB19009), Health and Family Planning Commission of Jiangxi Province (No. 2016A009), Science and Technology Project of Jiangxi Province (No. 20161BBH80002), Nanchang Innovative Talent Team (No. [2016]173), Education Department of Jiangxi Province (No.GJJ180669).

## Conflict of Interest

The authors declare that the research was conducted in the absence of any commercial or financial relationships that could be construed as a potential conflict of interest.

## Supplementary Material

Figure S1 Extraction protocols of GU water extract. (GE = Gastrodia, UR = Uncaria, h = hour, min = minute).

Figure S2 Base peak intensity (BPI) chromatograms of ten plasma QC sample injections: **(A)** for negative ion mode; **(B)** for positive ion mode.

Figure S3 Base peak intensity (BPI) chromatograms of ten intestinal lavage fluid QC sample injections: **(A)** for negative ion mode; **(B)** for positive ion mode.

Figure S4 Overview of permutations test plot for OPLS-DA (200 tests) for rat plasma samples. **(A)** Permutations test of OPLS-DA in negative ion mode for Control and Model groups; **(B)** Permutations test of OPLS-DA in positive ion mode for Control and Model groups.

Figure S5 Base peak intensity (BPI) chromatograms of seven standards: **(A)** for negative ion mode; **(B)** for positive ion mode. (P10) L-tryptophan; (P12) glutamic acid; (P13) L-arginine; (P17) 3-indoxyl sulfate; (P27) L-tryptophan; (P30) L-phenylalanine; (P31) L-tyrosine; (P32) L-valine.

Figure S6 Overview of metabolic profile after GU treatment: **(A)** ESI+, PCA; **(B)** ESI+, OPLS-DA; **(C)** ESI-, PCA; **(D)** ESI-, OPLS-DA. **(E)** Permutations test plot for OPLS-DA (200 tests) in positive ion mode for rat plasma samples; **(F)** Permutations test plot for OPLS-DA (200 tests) in negative ion mode for rat plasma samples.

Figure S7 Expression of 24 plasma biomarkers in all groups, *P < 0.05, **P < 0.01 compared with the model group; #P < 0.05, ##P < 0.01 compared with the control group (Control = Control group; Model = Model group; GUW = GU administration of rats in model group).

Table S1 RSD of typical peaks of plasma QC sample (ESI-.n=10; ESI+,n=10).

Table S2 RSD of typical peaks of intestinal lavage fluid QC sample (ESI-.n=10; ESI+,n=10).
