# Supplementary figures and images for: Metabolomics and 16S rRNA Gene Sequencing Analyses of Changes in the Intestinal Flora and Biomarkers Induced by Gastrodia-Uncaria Treatment in a Rat Model of Chronic Migraine
Source: Front Pharmacol. 2019 Dec 17;10:1425. doi: 10.3389/fphar.2019.01425 (PMC6929670; doi:10.3389/fphar.2019.01425)

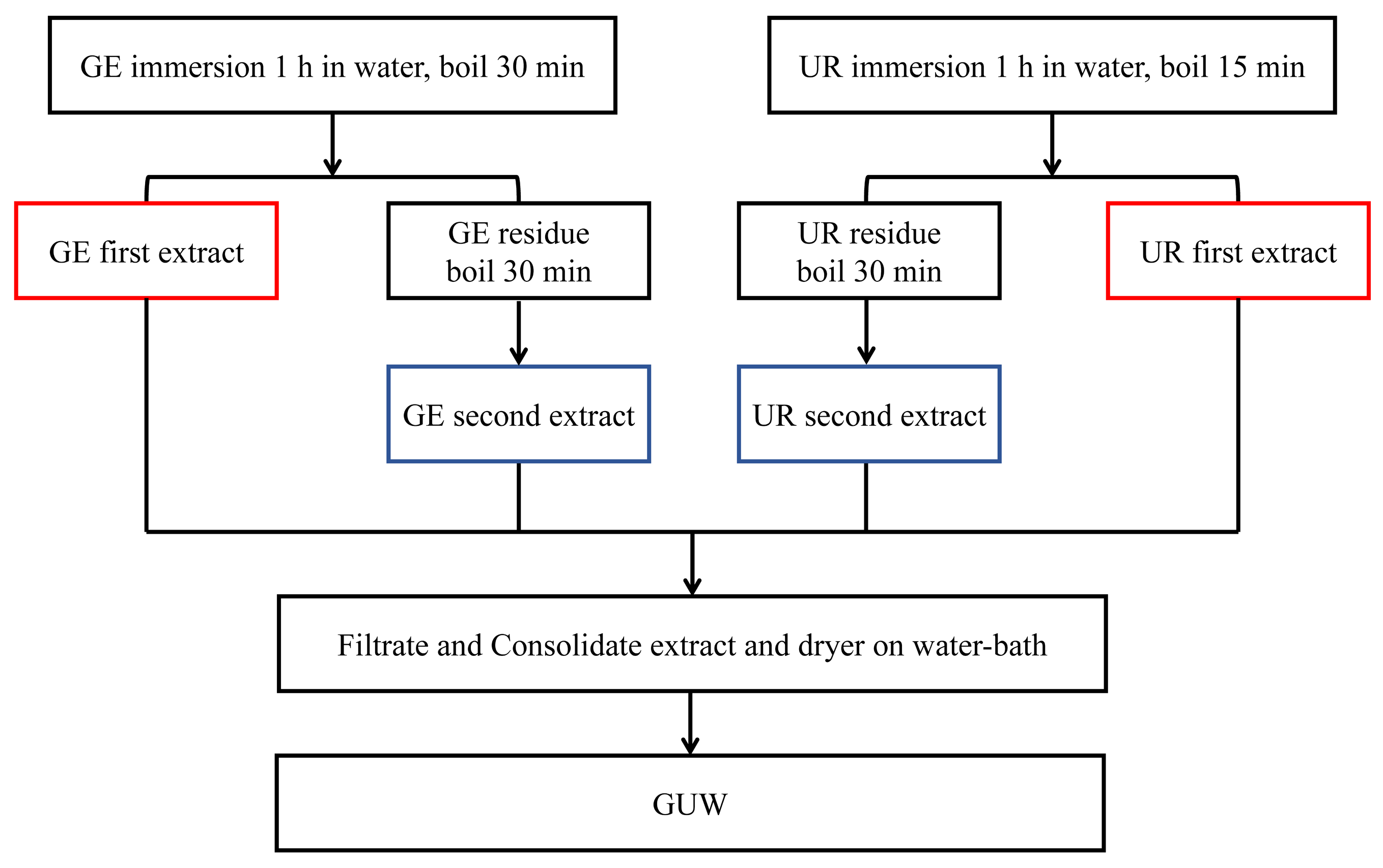

Supplement: Figure S1 — Extraction protocols of GU water extract. (GE = Gastrodia, UR = Uncaria, h = hour, min = minute). [file Image_1.tif]

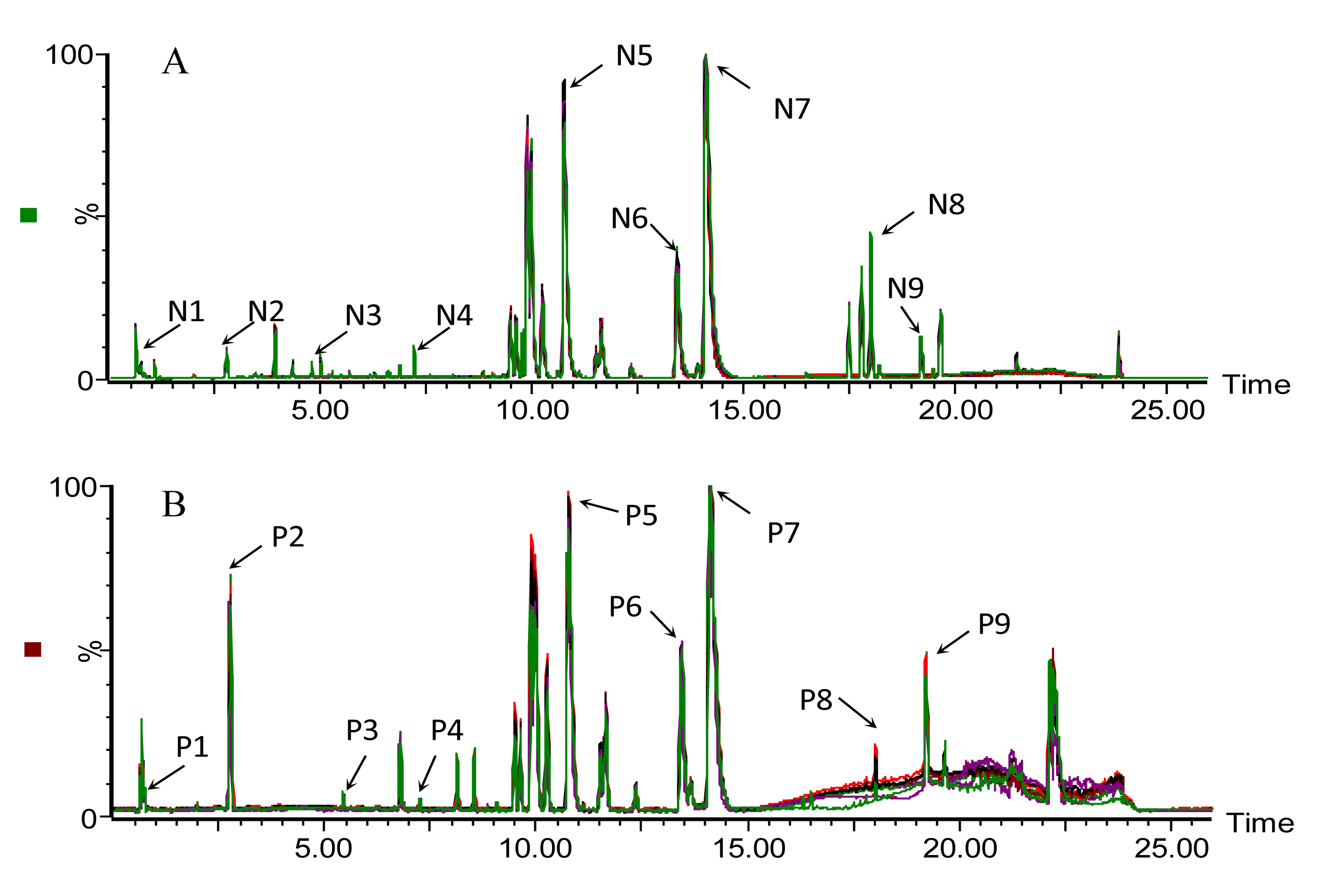

Supplement: Figure S2 — Base peak intensity (BPI) chromatograms of ten plasma QC sample injections: (A) for negative ion mode; (B) for positive ion mode. [file Image_2.tif]

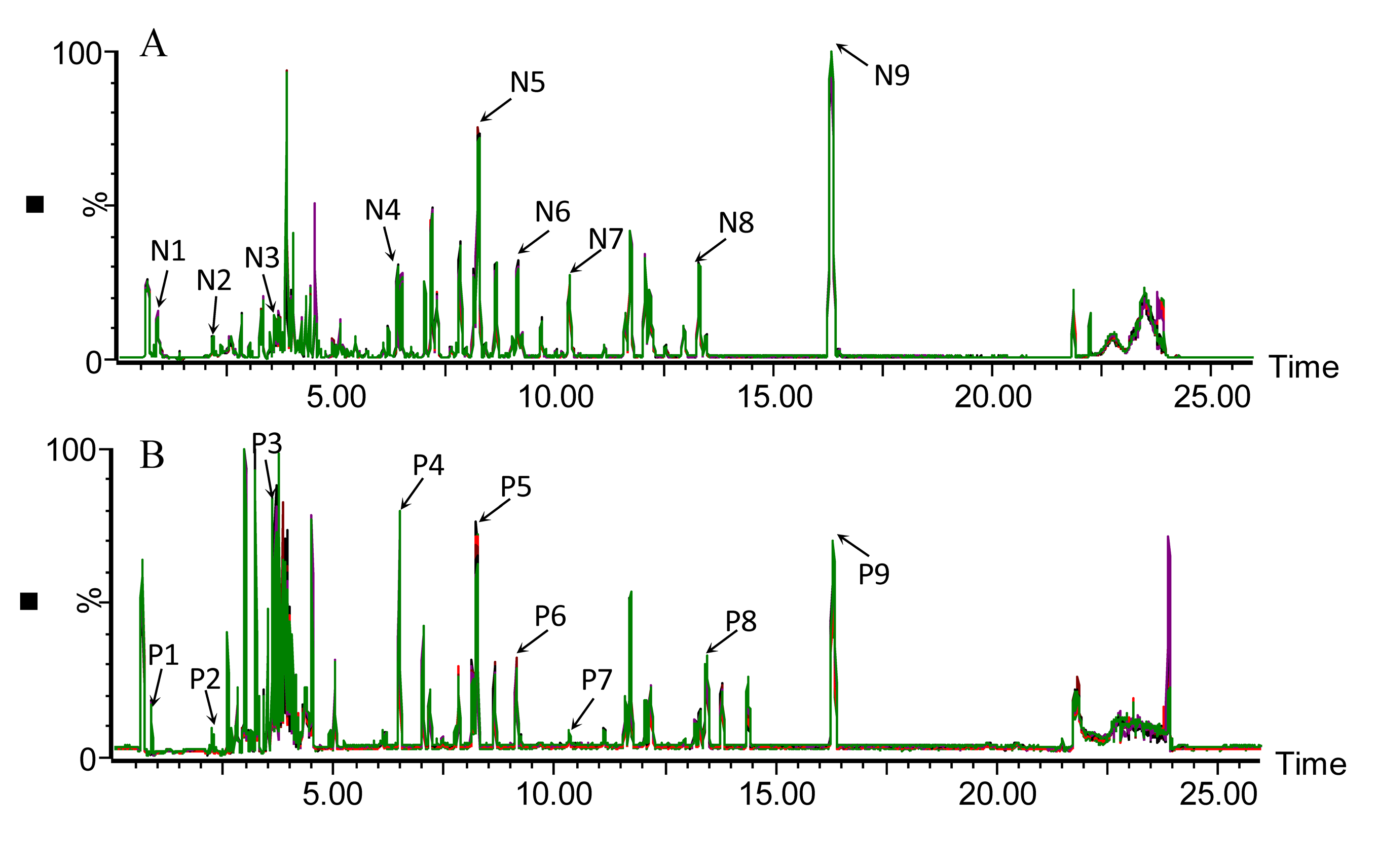

Supplement: Figure S3 — Base peak intensity (BPI) chromatograms of ten intestinal lavage fluid QC sample injections: (A) for negative ion mode; (B) for positive ion mode. [file Image_3.tif]

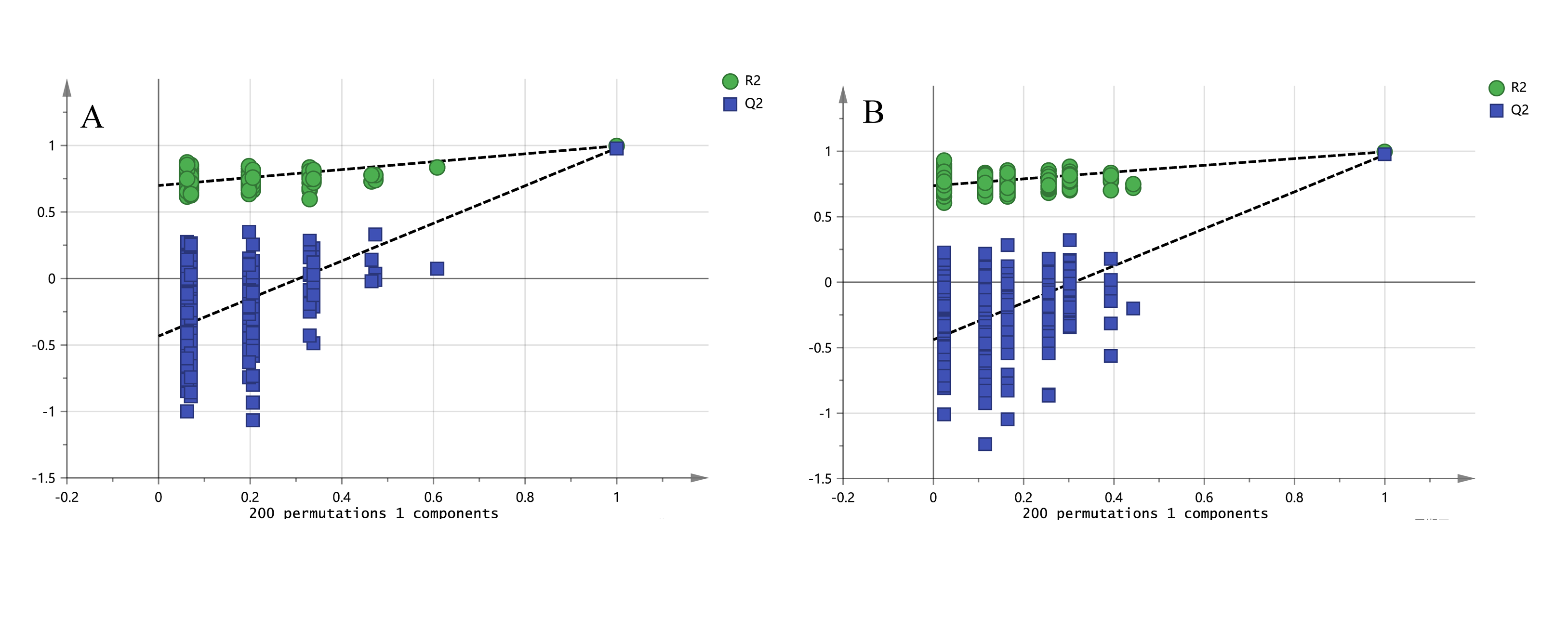

Supplement: Figure S4 — Overview of permutations test plot for OPLS-DA (200 tests) for rat plasma samples. (A) Permutations test of OPLS-DA in negative ion mode for Control and Model groups; (B) Permutations test of OPLS-DA in positive ion mode for Control and Model groups. [file Image_4.tif]

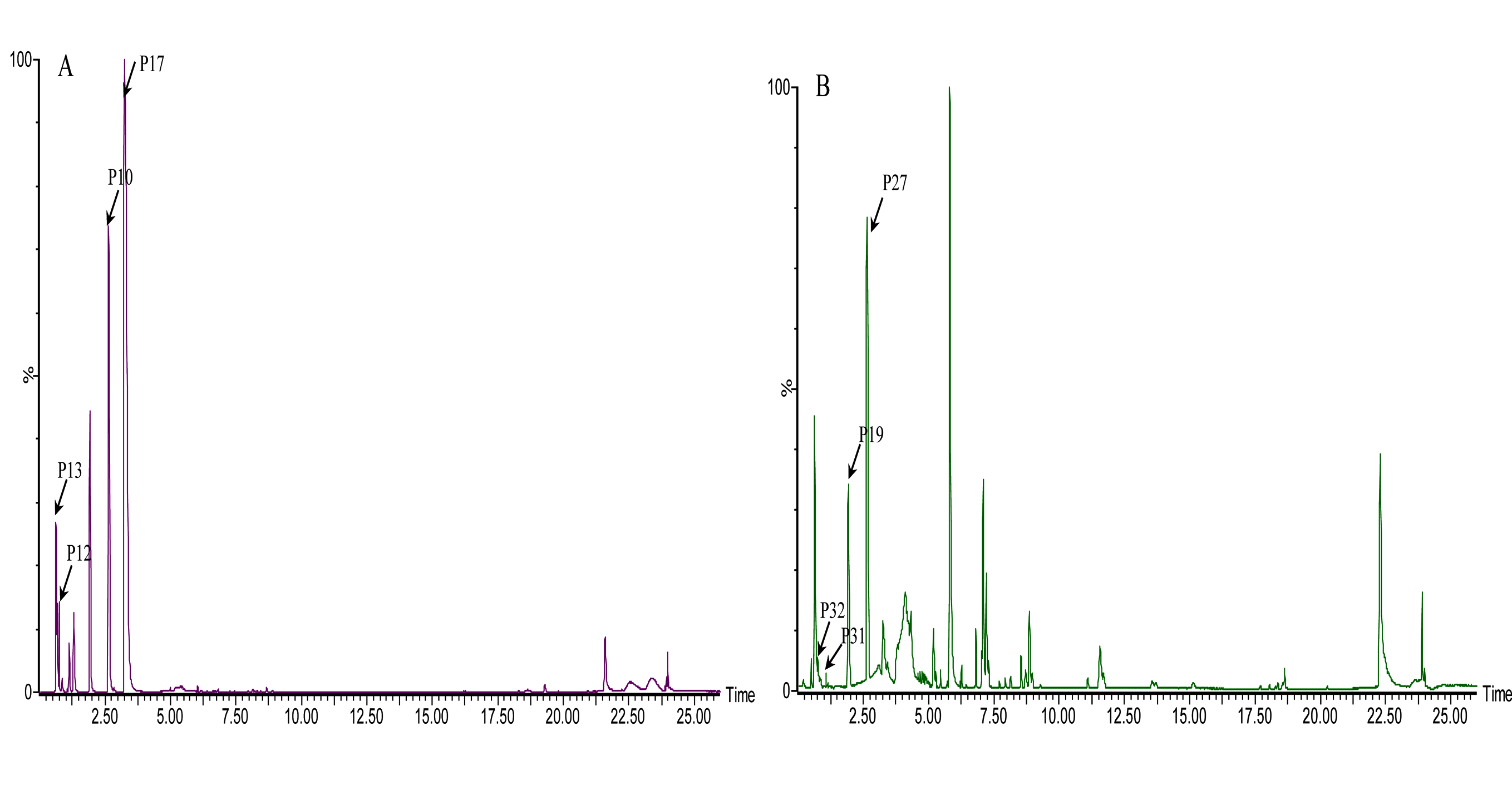

Supplement: Figure S5 — Base peak intensity (BPI) chromatograms of seven standards: (A) for negative ion mode; (B) for positive ion mode. (P10) L-tryptophan; (P12) glutamic acid; (P13) L-arginine; (P17) 3-indoxyl sulfate; (P27) L-tryptophan; (P30) L-phenylalanine; (P31) L-tyrosine; (P32) L-valine. [file Image_5.tif]

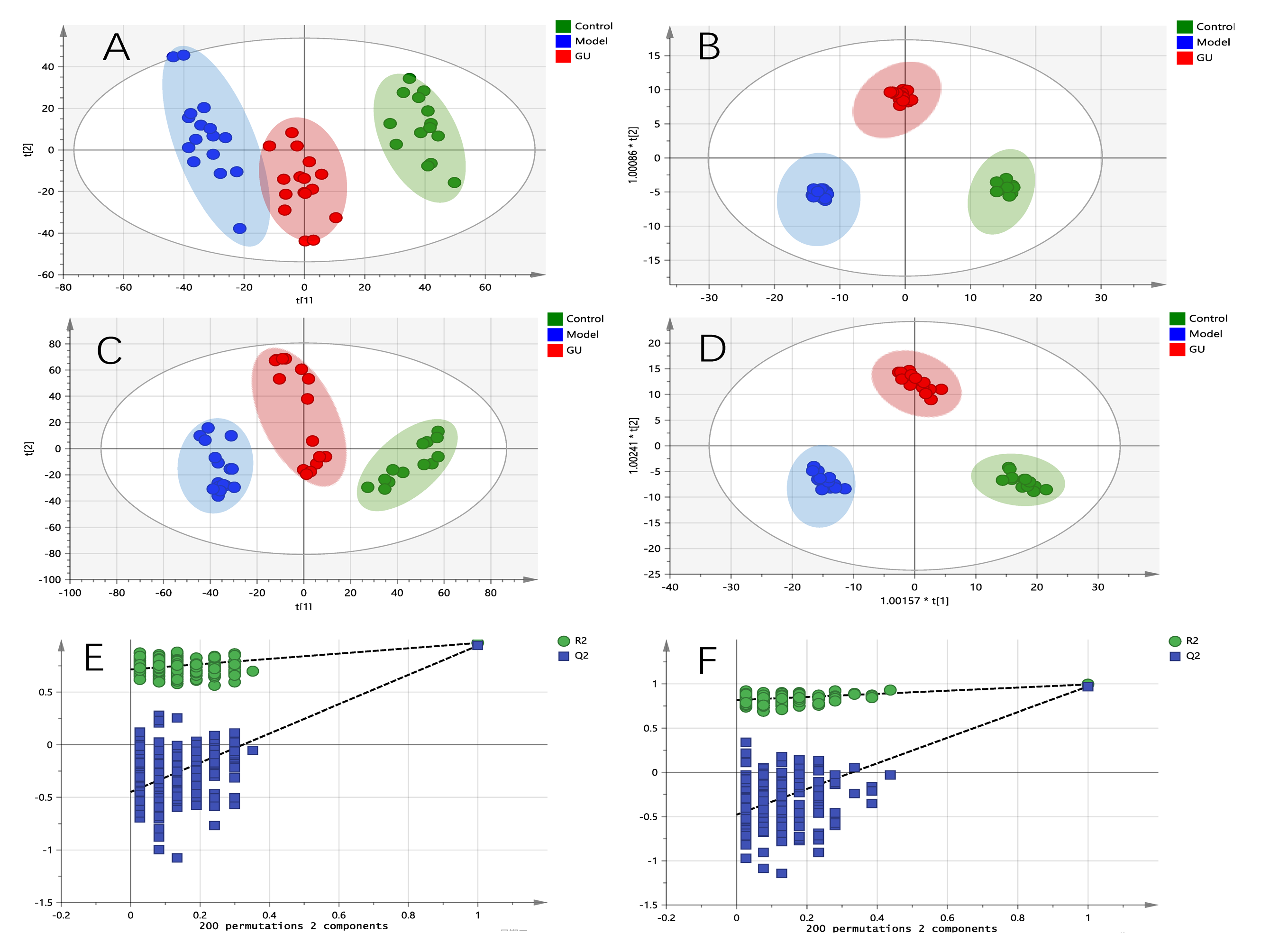

Supplement: Figure S6 — Overview of metabolic profile after GU treatment: (A) ESI+, PCA; (B) ESI+, OPLS-DA; (C) ESI-, PCA; (D) ESI-, OPLS-DA. (E) Permutations test plot for OPLS-DA (200 tests) in positive ion mode for rat plasma samples; (F) Permutations test plot for OPLS-DA (200 tests) in negative ion mode for rat plasma samples. [file Image_6.tif]

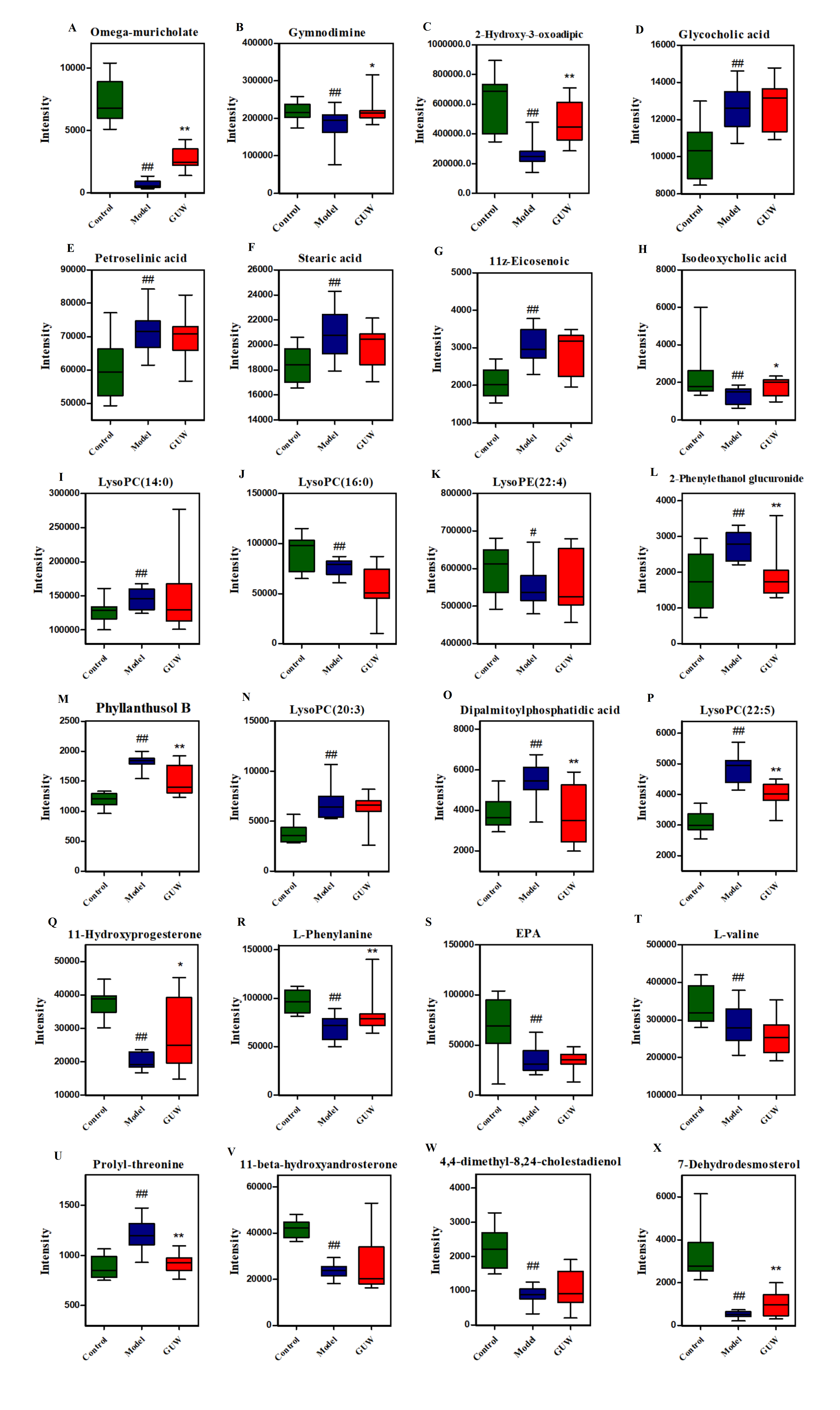

Supplement: Figure S7 — Expression of 24 plasma biomarkers in all groups, *P < 0.05, **P < 0.01 compared with the model group; #P < 0.05, ##P < 0.01 compared with the control group (Control = Control group; Model = Model group; GUW = GU administration of rats in model group). [file Image_7.tif]
