# Supplementary material for: Metabolomics and 16S rRNA Gene Sequencing Analyses of Changes in the Intestinal Flora and Biomarkers Induced by Gastrodia-Uncaria Treatment in a Rat Model of Chronic Migraine
Source: Front Pharmacol. 2019 Dec 17;10:1425. doi: 10.3389/fphar.2019.01425 (PMC6929670; doi:10.3389/fphar.2019.01425)
Supplement: Table S1 — RSD of typical peaks of plasma QC sample (ESI-.n=10; ESI+,n=10). [file Table_1.docx]

Table S1 RSD of typical peaks of plasma QC sample (ESI-.n=10; ESI+,n=10)

| **Peak name** | **MS** | **Ion mode** | **RSD** |
| --- | --- | --- | --- |
|  |  |  | **Intensity** |
| N1 | 238.9313 | ESI- | 5.88% |
| N2 | 198.0323 | ESI- | 6.95% |
| N3 | 344.0850 | ESI- | 6.77% |
| N4 | 448.0367 | ESI- | 4.75% |
| N5 | 566.3465 | ESI- | 6.80% |
| N6 | 480.3095 | ESI- | 6.97% |
| N7 | 594.3772 | ESI- | 10.66% |
| N8 | 329.2484 | ESI- | 8.38% |
| N9 | 646.8560 | ESI- | 15.79% |
| P1 | 226.9610 | ESI+ | 14.87% |
| P2 | 200.0473 | ESI+ | 10.65% |
| P3 | 185.1226 | ESI+ | 18.02% |
| P4 | 431.2928 | ESI+ | 7.60% |
| P5 | 496.3653 | ESI+ | 12.64% |
| P6 | 548.3926 | ESI+ | 5.34% |
| P7 | 546.3783 | ESI+ | 11.0% |
| P8 | 406.3448 | ESI+ | 18.16% |
| P9 | 749.5661 | ESI+ | 19.81% |
