# Supplementary material for: Metabolomics and 16S rRNA Gene Sequencing Analyses of Changes in the Intestinal Flora and Biomarkers Induced by Gastrodia-Uncaria Treatment in a Rat Model of Chronic Migraine
Source: Front Pharmacol. 2019 Dec 17;10:1425. doi: 10.3389/fphar.2019.01425 (PMC6929670; doi:10.3389/fphar.2019.01425)
Supplement: Table S2 — RSD of typical peaks of intestinal lavage fluid QC sample (ESI-.n=10; ESI+,n=10). [file Table_2.docx]

Table S2 RSD of typical peaks of intestinal lavage fluid QC sample (ESI-.n=10; ESI+,n=10)

| **Peak name** | **MS** | **Ion mode** | **RSD** |
| --- | --- | --- | --- |
|  |  |  | **Intensity** |
| N1 | 191.0566 | ESI- | 4.04% |
| N2 | 496.0482 | ESI- | 3.85% |
| N3 | 198.0322 | ESI- | 5.04% |
| N4 | 514.2908 | ESI- | 12.58% |
| N5 | 407.2798 | ESI- | 4.68% |
| N6 | 475.2670 | ESI- | 6.10% |
| N7 | 471.2417 | ESI- | 6.31% |
| N8 | 391.2844 | ESI- | 4.21% |
| N9 | 821.5313 | ESI- | 9.88% |
| P1 | 351.1061 | ESI+ | 10.18% |
| P2 | 311.1199 | ESI+ | 11.85% |
| P3 | 200.0477 | ESI+ | 4.80% |
| P4 | 628.2506 | ESI+ | 4.42% |
| P5 | 488.2986 | ESI+ | 10.07% |
| P6 | 431.2768 | ESI+ | 3.06% |
| P7 | 317.2476 | ESI+ | 6.18% |
| P8 | 877.1139 | ESI+ | 5.51% |
| P9 | 339.2683 | ESI+ | 1.85% |
